# Computational Prediction of Ammonia-Borane Dehydrocoupling and Transfer Hydrogenation of Ketones and Imines Catalyzed by SCS Nickel Pincer Complexes

**DOI:** 10.3389/fchem.2019.00627

**Published:** 2019-09-13

**Authors:** Bing Qiu, Wan Wang, Xinzheng Yang

**Affiliations:** ^1^Beijing National Laboratory for Molecular Sciences, State Key Laboratory for Structural Chemistry of Unstable and Stable Species, CAS Research/Education Center for Excellence in Molecular Sciences, Institute of Chemistry Chinese Academy of Sciences, Beijing, China; ^2^University of Chinese Academy of Sciences, Beijing, China

**Keywords:** lactate racemase, SCS nickel pincer, ammonia-borane, density functional theory, transfer hydrogenation, ketones, amines

## Abstract

Inspired by the catalytic mechanism and active site structure of lactate racemase, three scorpion-like SCS nickel pincer complexes were proposed as potential catalysts for transfer hydrogenation of ketones and imines with ammonia-borane (AB) as the hydrogen source. Density functional theory calculations reveal a stepwise hydride and proton transfer mechanism for the dehydrocoupling of AB and hydrogenation of N-methylacetonimine, and a concerted proton-coupled hydride transfer process for hydrogenation of acetone, acetophenone, and 3-methyl-2-butanone. Among all proposed Ni complexes, the one with symmetric NH_2_ group on both arms of the SCS pincer ligand has the lowest free energy barrier of 15.0 kcal/mol for dehydrogenation of AB, as well as total free energy barriers of 17.8, 18.2, 18.0, and 18.6 kcal/mol for hydrogenation of acetone, N-methylacetonimine, acetophenone, and 3-methyl-2-butanone, respectively.

## Introduction

Compared with direct hydrogenation, transfer hydrogenation (TH) avoids the use of hazardous molecular hydrogen and high pressure equipment by adopting low-cost and safe hydrogen provider compounds (Gladiali and Alberico, 2006; Ikariya and Blacker, 2007). Transition-metal-catalyzed TH has attracted increasing attentions in pharmaceutical, agrochemical, fragrance and other fine chemical industries as a powerful and practical way for the production of valuable chiral alcohols and amines (Blaser et al., 2003, 2007; Klingler, 2007; Saudan, 2007; Hansen et al., 2009).

The first TH reaction is Meerwein-Pondorf-Verley (MPV) reduction of ketone reported in mid-1920s (Meerwein and Schmidt, 1925; Verley, 1925; Ponndorf, 1926). In MPV reduction, direct TH happens through a cyclic six-membered transition state with alcohol and the carbonyl coordinated to the aluminum center. However, the low enantio-selectivity and undesired side reactions are well-known drawbacks in MPV reduction. In early 1980s, Matteoli et al. (1981) reported the first catalytic asymmetric transfer hydrogenation (ATH) reaction, in which a ruthenium complex H_4_Ru_4_(CO)_8_[(–)-DIOP]_2_ was used as the catalyst with secondary alcohols or indoline as the hydrogen source, for hydrogenation of prochiral ketones. To date, although significant progress has been made in transition metal-catalyzed TH and ATH reactions (Gopalaiah, 2013; Zuo et al., 2013, 2016; Pellissier and Clavier, 2014; Li et al., 2015; Morris, 2015; Wang and Astruc, 2015; Zuo and Morris, 2015), most reported catalysts are based on expensive and toxic noble metals, such as Rh, Ir, Ru, etc. (Gopalaiah, 2013; Pellissier and Clavier, 2014; Li et al., 2015; Morris, 2015; Wang and Astruc, 2015). The replacement of high-cost and toxic precious metals with abundant and environmentally benign base metals in catalysts for efficient TH and ATH reactions has attracted increasing attention in recent years, and several iron catalysts have been reported (Zhou et al., 2011; Gopalaiah, 2013; Zuo et al., 2013, 2016; Pellissier and Clavier, 2014; Li et al., 2015; Lu et al., 2015; Morris, 2015; Zuo and Morris, 2015; Smith et al., 2017). For example, Gao (Li et al., 2015) and Morris (Zuo et al., 2013; Morris, 2015; Zuo and Morris, 2015) groups reported tetradentate PNNP iron catalysts for ATH of acetophenone with high enantioselectivities. Morris and co-workers (Smith et al., 2017) reported unsymmetrical iron P-NH-P' complexes for asymmetric hydrogenation of aryl ketones with *ee* values >90%. Morris (Zuo et al., 2013; Morris, 2015) and Beller (Zhou et al., 2011; Lu et al., 2015) groups developed iron catalyzed asymmetric hydrogenation of imines and found high enantioselectivities for the reductions of N-phosphinyl ketimines.

In contrast to the encouraging progress archived in iron catalysts, ATH of ketones catalyzed by cobalt complexes have rather low enantioselectivities (Morris, 2015). Only a few Ni catalysts have been reported so far (Hamada et al., 2008; Hibino et al., 2009; Dong et al., 2012; Yang et al., 2014; Xu et al., 2015). In 2008, Hamada and co-workers (Hamada et al., 2008) applied nickel-bisphosphine complexes to catalyze asymmetric hydrogenation of α-amino-β-keto ester hydrochlorides and achieved high diastereo- and enantioselectivities (88–93% *ee*) for the production of *anti*-β-hydroxy-α-amino esters. They also used the same catalyst for asymmetric hydrogenation of substituted aromatic α-aminoketone hydrochlorides to produce β-aminoalcohols and found excellent diastereo- and enantioselectivities (Hibino et al., 2009). In 2012, Dong et al. (2012) reported Ni(II) complexes chelated by PNO ligands for catalytic ATH of aromatic ketones with 2-propanol as the hydrogen source and obtained optical alcohols up to 84% *ee* under mild conditions. In 2014, Yang et al. (2014) reported a highly active Ni(OAc)_2_/Binapine catalyst for the synthesis of α- and β-amino acid trough ATH of olefins with formic acid. Later on, they reported a highly active NiCl_2_(dme)/Binapine catalysts for ATH of hydrazones and other ketimines (Xu et al., 2015).

In a typical TH process, low-cost compounds such as 2-propanol (Haack et al., 1997) and formic acid (Fujii et al., 1996), are used as hydrogen donors. However, reversibility is a major drawback while 2-propanol is used as the hydrogen source. Excessive 2-propanol are usually required for the formation of alcohol. Formic acid releases stable CO_2_ after its dehydrogenation and does not suffer from equilibrium problems but can only be used for a limited range of stable complexes. Compared to alcohols, amine-boranes are easy to handle, irreversible, and potentially recyclable hydrogen donors (Nixon et al., 2011). Therefore, amine-boranes, especially ammonia-borane (AB), are promising to serve as solid hydrogen surrogates in TH reactions (Nixon et al., 2011). In recent years, Pagano et al. (2015) reported cobalt catalyzed dehydrocoupling of AB and TH of alkenes and alkynes. Fu et al. (2016), Shao et al. (2016), and Ai et al. (2019) reported the first cobalt-catalyzed selective TH of alkynes, and the only example of cobalt-catalyzed TH of nitriles with dehydrocoupling of AB under mild conditions. Yang et al. (2010, 2011a,b) recently reported direct TH of polarized C=N, C=C, and C=O bonds in imines, olefins, ketones, and aldehydes from AB. Li et al. (2016, 2017), Meng et al. (2018) reported frustrated Lewis pair catalyzed ATH of imines with AB and ATH of 2,3-disubstituted quinoxalines with AB. Ding et al. (2017) reported TH of N-heterocycles from AB prompted by B(C_6_F_5_)_3_. Korytiakova et al. (2017) reported Cu(I) catalyzed transfer semihydrogenation of alkyne and conjugate transfer hydrogenation of enoates with the dehydrocoupling of AB. Barrios-Francisco and García (2010) reported semihydrogenation of alkynes from AB catalyzed by Ni(0) complexes. Chong et al. (2014) reported 1,3,2-diazaphospholenes-catalyzed metal-free TH of N=N double bond using AB. Although some iron and nickel catalysts for acceptorless dehydrogenation of AB have also been developed (Keaton et al., 2007; Yang and Hall, 2008; Zimmerman et al., 2009a,b; Vogt et al., 2011; Bhunya et al., 2016; Lunsford et al., 2016; Rossin and Peruzzini, 2016; Chakraborty et al., 2017; Coles et al., 2017), effective base metal catalyst for TH of ketones using AB under mild conditions was rarely reported.

Inspired by the catalytic mechanism and the active site structure of lactate racemase (Desguin et al., 2015), as well as previously reported SCS nickel pincer complexes (Meguro et al., 2008; Xu et al., 2017), we recently proposed and computationally predicted several promising scorpion-like SCS nickel pincer complexes for catalytic lactate racemization and ATH of 1-acetonaphthone based on density functional theory (DFT) calculations (Qiu and Yang, 2017; Qiu et al., 2019). In this paper, we further examined three SCS nickel pincer complexes as potential catalysts for fast dehydrocoupling of AB and TH of ketones and imines.

## Results and Discussion

**Figure 1:**
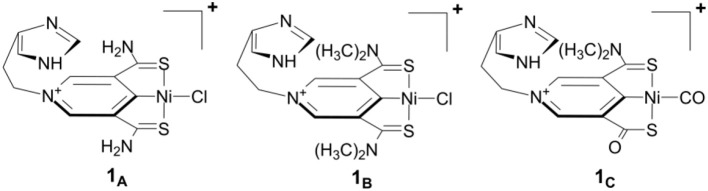
Scorpion-like (SCS)Ni pincer complexes proposed by Qiu and Yang (2017).

Figure 1 shows our previously proposed scorpion-like SCS nickel pincer complexes **1**_A_, **1**_B_, and **1**_C_ with imidazole tails. **1**_A_ and **1**_B_ have symmetric arms but different sizes of amino groups in the SCS ligand, which are similar to the Ni complexes with pincer SCS ligands synthesized by Hu and co-workers (Xu et al., 2017). **1**_C_ is a mimic of the active site of lactate racemase with a carbonyl arm in the SCS pincer ligand.

**Scheme 1:**
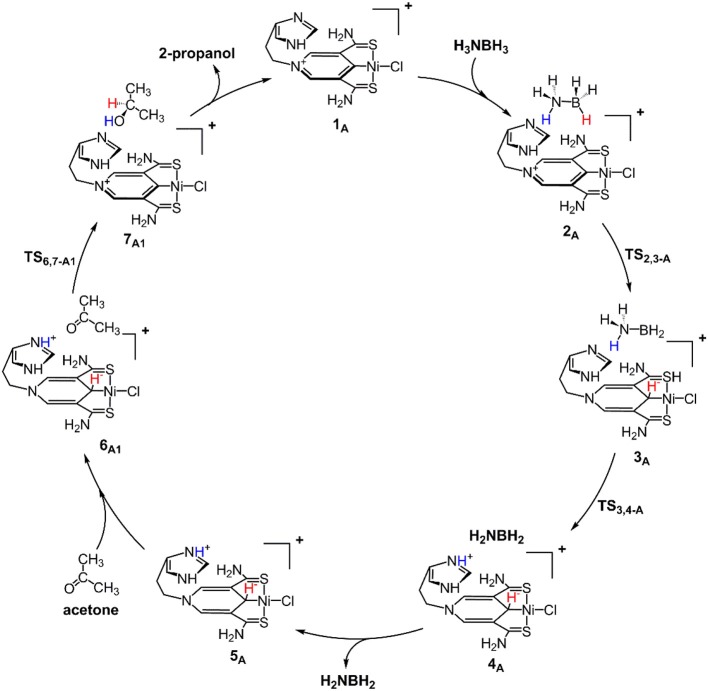
Proposed mechanism for the dehydrocoupling of AB and TH of acetone catalyzed by **1**_A_.

**Figure 2:**
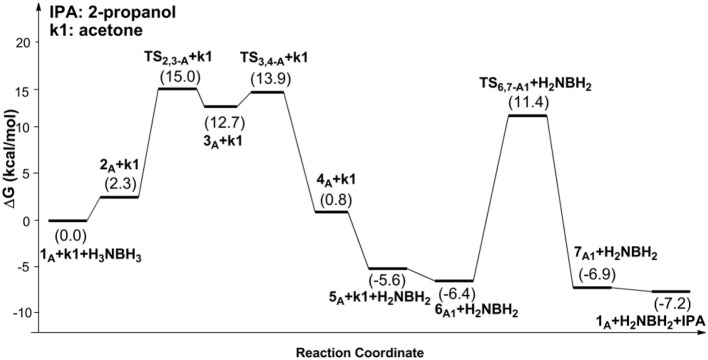
Free energy profile for the dehydrocoupling of AB and TH of acetone catalyzed by **1**_A_.

**Figure 3:**
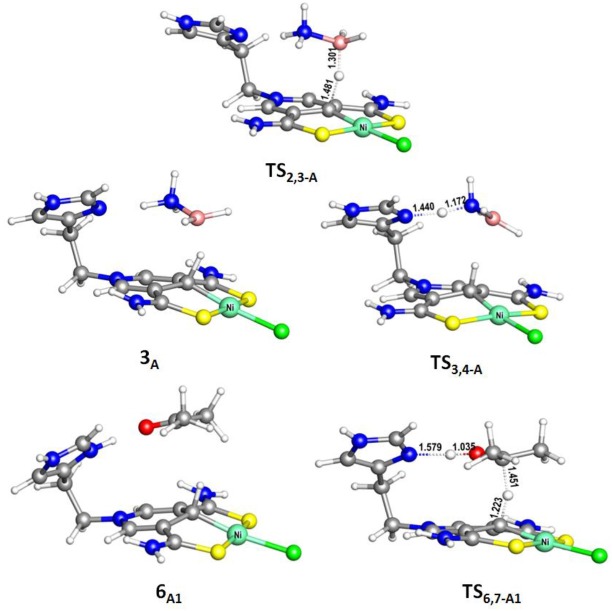
Optimized structures of **6**_A1_, **TS**_2,3−A_ (495*i* cm^−1^), **TS**_3,4−A_ (388*i* cm^−1^), and **TS**_6,7−A1_ (510*i* cm^−1^). Bond lengths are in angstrom.

**Scheme 2:**
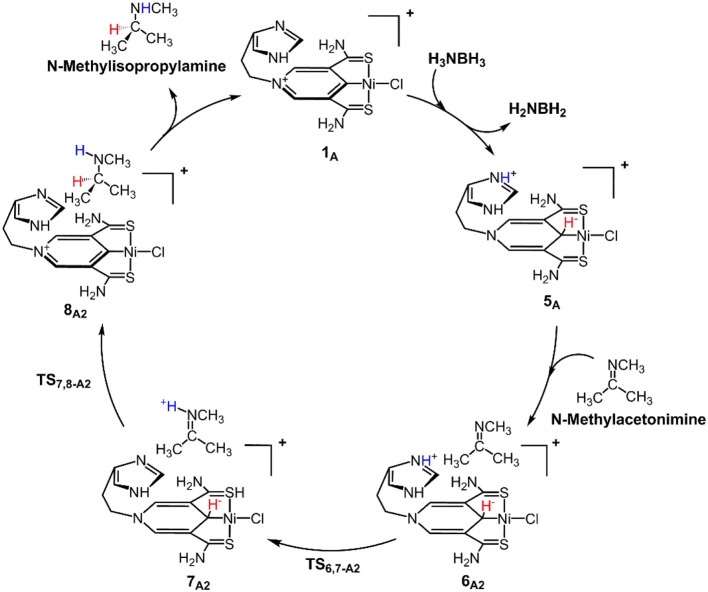
**1**_A_ catalyzed TH of N-methylisopropylamine with stepwise proton and hydride transfers.

**Figure 4:**
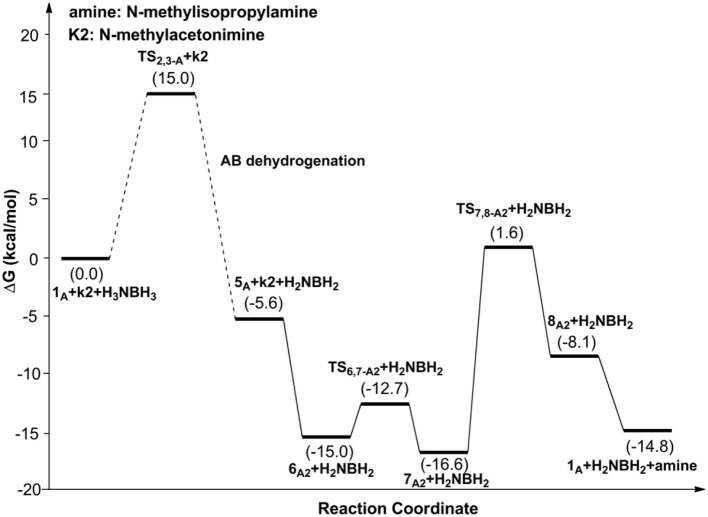
Free energy profile for the TH of N-methylacetonimine catalyzed by **1**_A_.

**Figure 5:**
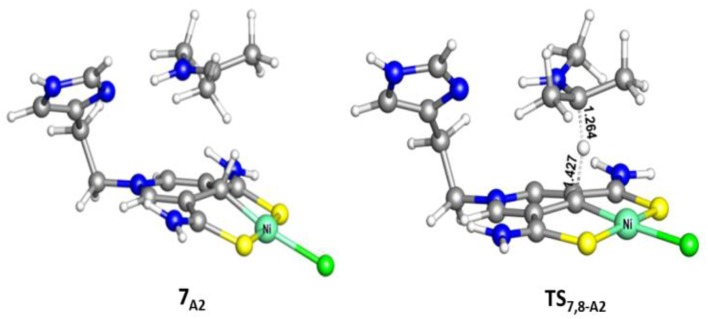
Optimized structures of **7**_**A2**_ and **TS**_**7,8−A2**_ (700i cm^−1^). Bond lengths are in angstrom.

Scheme 1 is the proposed reaction cycle for the dehydrocoupling of AB and hydrogenation of acetone to 2-propanol catalyzed by **1**_A_. Figure 2 reports the corresponding free energy profile. The optimized structures of key intermediates and transition states in the reaction are displayed in Figure 3. The reaction cycle, free energy profile, and key structures of the TH of N-methylacetonimine catalyzed by **1**_A_are shown in Scheme 2, Figures 4, 5, respectively.

The transfer hydrogenation reaction starts with dehydrocoupling of AB. When an AB molecule approaches **1**_A_, a hydride on borane could be transferred to the sp^2^ carbon coordinated to nickel through transition state **TS**_2,3−A_ (ΔG = 15.0 kcal/mol) and forms an unstable intermediate **3**_A_. An ammonia proton then transfers to the imidazole nitrogen through transition state **TS**_3,4−A_ (ΔG = 13.9 kcal/mol). The exchange of acetone and H_2_NBH_2_ in **4**_A_ forms a 7.2 kcal/mol stable intermediate **6**_A1_. Then the proton and hydride obtained from AB transfer from the pincer ligand in **6**_A1_ to acetone in one-step through **TS**_6,7−A1_ (Figure 3) with a free energy barrier of 17.8 kcal/mol for the formation of 2-propanol.

Different from the hydrogenation of acetone, the proton and hydride are transferred from the pincer ligand in **6**_A2_ to N-methylacetonimine in a stepwise way with two transition states, **TS**_6,7−A2_ and **TS**_7,8−A2_. The total free energy barrier of the hydrogenation of N-methylacetonimine is 18.2 kcal/mol (**7**_A2_ → **TS**_7,8−A2_).

Table 1 lists calculated free energy barriers of the hydrogenations of acetone and N-methylacetonimine (ΔG_acetone_ and ΔG_N−methylacetonimine_) catalyzed by **1**_A_, **1**_B_, and **1**_C_. The difference of those relative free energies are less than 4 kcal/mol, which indicates a rather weak steric effect with different functional groups in the pincer ligand.

**Table 1:** Free energy barriers of the TH of acetone and N-methylacetonimine with AB catalyzed by **1**_A_, **1**_B_, and **1**_C_.

**Catalysts**	**ΔG_**acetone**_ (kcal/mol) (6_**X1**_ → TS_**6, 7-X1, X = A, B, C**_)**	**ΔG_**N-methylacetonimine**_ (kcal/mol) (7_**X2**_ → TS_**7, 8-X2, X = A, B, C**_)**
**1**_A_	17.8	18.2
**1**_B_	19.3	16.4
**1**_C_	20.0	19.2

**Figure 6:**
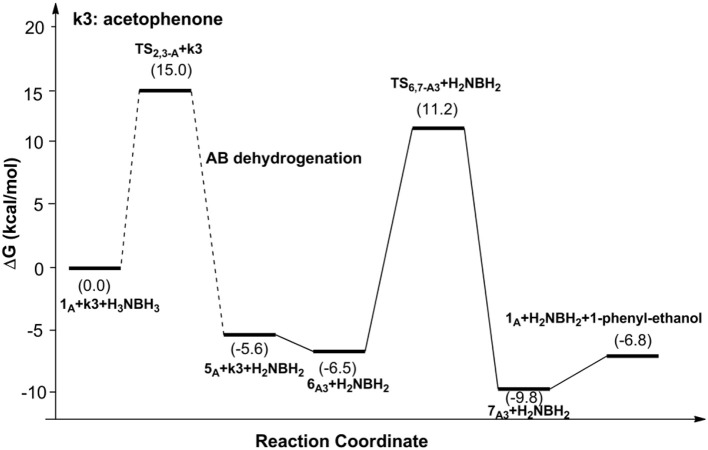
Free energy profile for the TH of acetophenone catalyzed by **1**_A_.

**Figure 7:**
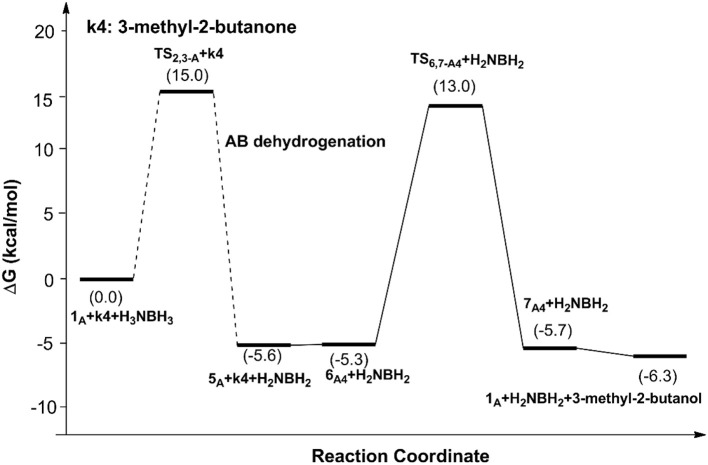
Free energy profile for the TH of 3-methyl-2-butanone catalyzed by **1**_A_.

**Figure 8:**
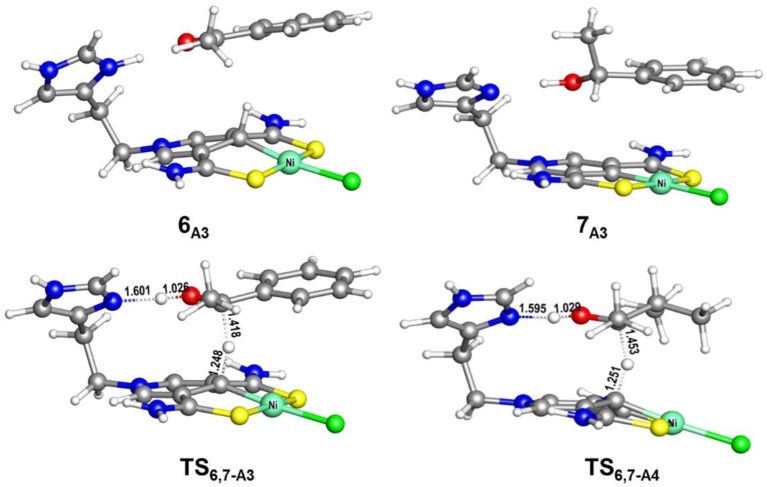
Optimized structures of **6**_A3_, **7**_A3_, **TS**_6,7−A3_ (485i cm^−1^), and **TS**_6,7−A4_ (663i cm^−1^). Bond lengths are in angstrom.

Figures 6, 7 report free energy profiles for **1**_A_ catalyzed TH of acetophenone to 1-phenyl-ethanol and TH of 3-methyl-2-butanone to 3-methyl-2-butanol, respectively. Some key structures in those catalytic reactions are displayed in Figure 8.

The AB dehydrocoupling process has the same free energy barrier of 15.0 kcal/mol in Figures 6, 7. The hydrogenation of acetophenone goes through a concerted one-step proton and hydride transfer transition state **TS**_6,7−A3_ (Figure 8). Although **TS**_6,7−A3_ is 17.7 kcal/mol higher than **7**_A3_, the total free energy barrier of this catalytic TH reaction is 18.0 kcal/mol (**7**_A3_ → **TS**_2,3−A_) after considering the barrier of AB dehydrogenation.

The THs of acetophenone and 3-methyl-2-butanone catalyzed by **1**_A_, **1**_B_, and **1**_C_ have similar mechanisms but slightly different energy barriers, which are listed in Table 2. We can see the free energy barriers of **1**_B_ and **1**_C_ are higher because of the stronger steric effects of dimethyl groups in them.

**Table 2:** Free energy barriers of the TH of acetophenone and 3-methyl-2-butanone with AB catalyzed by **1**_A_, **1**_B_, and **1**_C_.

**Catalysts**	**ΔG_**acetophenone**_ (kcal/mol)**	**ΔG_**3-methyl-2-butanone**_ (kcal/mol) (5_**X**_ → TS_**6, 7-X4, X = A, B, C**_)**
**1**_A_	18.0 (**7**_A3_ → **TS**_2,3−A_)	18.6
**1**_B_	19.2 (**7**_B3_ → **TS**_2,3−B_)	19.5
**1**_C_	19.7 (**6**_C3_ → **TS**_6,7−C3_)	20.8

It is worth to note that the role of the imidazole groups in those scorpion-like SCS nickel pincer complexes are proton reservoirs facilitating proton transfer in the dehydrocoupling of AB and hydrogenation of C=O and C=N bonds. The ethylene group connecting the pyridinium ring and the imidazole group ensures the adjustability of the imidazole group's position for the hydrogenation of different ketones and imines. The substituents on the arms of the SCS ligand can slightly influence the reaction barriers through their steric effects. We believe the stepwise hydride and proton transfers in hydrogenation of 3-methyl-2-butanone are caused by the weak polarity of the C=N bond in it.

## Conclusions

In summary, we computationally examined three scorpion-like SCS nickel pincer complexes, **1**_A_, **1**_B_, and **1**_C_, with different steric effects as potential catalysts for catalytic TH of ketones and imines. Our calculations reveal stepwise hydride and proton transfer processes for the dehydrocoupling of AB and hydrogenation of imine, and a proton coupled hydride transfer process for hydrogenation of ketones. Among three examined Ni complexes, **1**_A_ with symmetric NH_2_ groups in the pincer ligand has the lowest free energy barriers of 17.8, 18.2, 18.0, and 18.6 kcal/mol for transfer hydrogenations of acetone, N-methylacetonimine, acetophenone, and 3-methyl-2-butanone, respectively. **1**_B_ and **1**_C_ have slightly higher barriers for the same reactions because of their stronger steric effects. Such low barriers and exothermicities shown in calculated free energy profiles indicate that those (SCS)Ni pincer complexes are promising catalyst candidates for efficient TH of ketones and amines under mild conditions. The steric effects of the substituents on the arms of the pincer ligand have very weak influence on the energy barriers of the catalytic reactions. Our computational predictions not only provide prototypical base metal catalysts for dehydrocoupling of AB and transfer hydrogenation of ketones and imines, but also shed a light for further development of cost-effective catalysts for the hydrogenation of polarized double bonds. Further design of base metal complexes with SCS pincer ligands for more hydrogenation and dehydrogenation reactions is underway.

## Computational Details

The Gaussian 09 suite of ab initio programs (Frisch et al., 2010) was employed to perform all DFT calculations at the ultrafine (99,590) numerical integration level for the ωB97X-D (Chai and Head-Gordon, 2008) functional with Stuttgart relativistic effective core potential (ECP10MDF) basis set (Martin and Sundermann, 2001) and all-electron 6-31+G(d,p) basis set (Hehre et al., 1972; Hariharan and Pople, 1973; Francl et al., 1982) for Ni and all other atoms, respectively. Without other specific instruction, all structures in this paper were fully optimized in THF by using the integral equation formalism polarizable continuum model (IEFPCM) (Tomasi et al., 2005) with SMD (Marenich et al., 2009) atomic radii solvent corrections. The ground states of all structures were confirmed as singlet through comparison with optimized high-spin analogs. Thermal corrections were considered under 298.15 K and 1 atm pressure through frequency calculations using the same method on optimized structures. The optimized structures were confirmed to have no imaginary vibrational mode for intermediates and only one imaginary vibrational mode for each transition state, which was further confirmed by intrinsic reaction coordinate (IRC) calculations to ensure proper stationary points were connected. The 3D molecular structures displayed in the text were drawn by using the JIMP2 program (Manson et al., 2006). We also evaluated the performance of various density functionals and the influence of solvent effect corrections for this Ni catalytic system. The calculation results and discussions are provided in the Supplementary Materials (Tables S1 and S2).

## Data Availability

All datasets generated for this study are included in the manuscript and/or the Supplementary Files.

## Author Contributions

XY proposed the computational catalyst design project and the catalytic transfer hydrogenation reactions. BQ designed and computed all of the catalysts with the help of XY. BQ and WW wrote the paper with the help of XY.

### Conflict of Interest Statement

The authors declare that the research was conducted in the absence of any commercial or financial relationships that could be construed as a potential conflict of interest.
