# Supplementary material for: Computational Prediction of Ammonia-Borane Dehydrocoupling and Transfer Hydrogenation of Ketones and Imines Catalyzed by SCS Nickel Pincer Complexes
Source: Front Chem. 2019 Sep 13;7:627. doi: 10.3389/fchem.2019.00627 (PMC6753508; doi:10.3389/fchem.2019.00627)
Supplement: Supplementary file 1 [file Data_Sheet_1.PDF]

# *Supplementary Material*

## 1 Evaluation of density functionals

In order to examine the reliability of density functionals for this Ni system, we also calculated the free energy gaps between key intermediates and transition states using other six density functionals with different percentages of Hartree-Fock exchange, including B3LYP,[1-2] B3LYP-D3(BJ)[3], M06,[4] M06-2X,[4] TPSS,[5] and *ω*B97X.[6] The computed free energy gaps are listed in **Table S1**. All structures were optimized independently using the above functionals with the same basis set described in Computational details. We can see that the functionals without dispersion corrections, such as B3LYP, TPSS and *ω*B97X, have much larger relative free energies. Such a strong influence of the dispersion correction is not unexpected because this (SCS)Ni system has some non-covalent interactions in the reaction. The difference of the ΔG values obtained by using the M06, M06-2X and *ω*B97X-D functionals are less than 1.0 kcal/mol, which indicates that the (SCS)Ni system has a weak dependency of the density functionals. Therefore, we believe *ω*B97X-D is a suitable functional for the study of this (SCS)Ni system and our computational predictions are reliable.

**Table S1.** Relative free energies in the reactions catalyzed by $\mathbf{1_A}$ calculated by using different functionals. All other computational details are described in the text.

| Functionals | ΔG (kcal/mol) ($\mathbf{6_{A1}} \rightarrow \mathbf{TS_{6,7\text{-}A1}}$) | ΔG (kcal/mol) ($\mathbf{7_{A2}} \rightarrow \mathbf{TS_{7,8\text{-}A2}}$) | ΔG (kcal/mol) ($\mathbf{5_{A}} \rightarrow \mathbf{TS_{6,7\text{-}A3}}$) | ΔG (kcal/mol) ($\mathbf{5_{A}} \rightarrow \mathbf{TS_{6,7\text{-}A4}}$) |
|---|---|---|---|---|
| B3LYP | 24.4 | 23.1 | 31.2 | 33.7 |
| B3LYP-D3(BJ) | 13.3 | 13.6 | 12.1 | 15.3 |
| M06 | 16.9 | 15.6 | 17.3 | 18.8 |
| M06-2X | 16.3 | 17.3 | 17.7 | 18.9 |
| TPSS | 18.8 | 16.3 | 24.9 | 27.2 |
| *ω*B97X | 24.1 | 22.0 | 26.2 | 27.4 |
| *ω*B97X-D | 17.8 | 18.2 | 16.8 | 18.6 |

## 2 Evaluation of solvent effect

The calculated relative free energies of in the reactions catalyzed by $\mathbf{1_A}$ in water, THF and acetonitrile are listed in **Table S2**. We can see different solvents have very weak influence to the relative energies.

**Table S2.** Relative free energies in the reactions catalyzed by $\mathbf{1_A}$ calculated by using different solvents.

| Solvent | ΔG (kcal/mol) ($\mathbf{6_{A1}} \rightarrow \mathbf{TS_{6,7\text{-}A1}}$) | ΔG (kcal/mol) ($\mathbf{7_{A2}} \rightarrow \mathbf{TS_{7,8\text{-}A2}}$) | ΔG (kcal/mol) ($\mathbf{6_{A3}} \rightarrow \mathbf{TS_{6,7\text{-}A3}}$) | ΔG (kcal/mol) ($\mathbf{5_{A}} \rightarrow \mathbf{TS_{6,7\text{-}A4}}$) |
|---|---|---|---|---|
| Water | 20.2 | 18.1 | 20.7 | 21.7 |
| Acetonitrile | 20.4 | 20.4 | 19.3 | 21.7 |
| THF | 17.8 | 18.2 | 17.7 | 18.6 |

## 3 Isomers and their relative energies of $\mathbf{1_A}$

As shown in **Figure S1**, $\mathbf{1_A}$ is the proposed catalyst with the imidazole group on top of the pyridinium ring. $\mathbf{1_A}$**'** is an isomer of $\mathbf{1_A}$ the imidazole group at a position far away from the metal center. $\mathbf{1_A}$**''** is an isomer of $\mathbf{1_A}$ the imidazole group at a position close to another direction**.** Calculation results indicate that $\mathbf{1_A}$ is 1.5 kcal/mol more stable than $\mathbf{1_A}$**'** and 2.5 kcal/mol more stable than $\mathbf{1_A}$**''.**

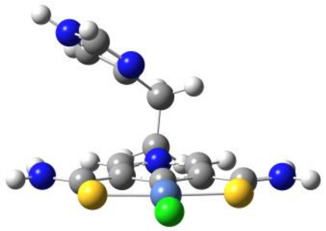

$\mathbf{1_A}$ ($G_{solv}$ = −2165.906340 a.u.)

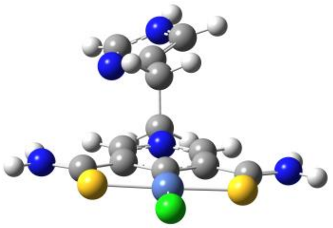

$\mathbf{1_A}$**'** ($G_{solv}$ = −2165.903951 a.u.)

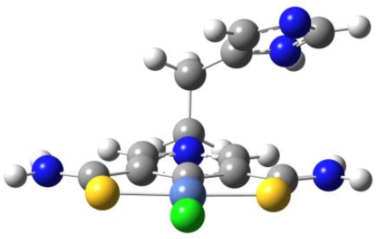

$\mathbf{1_A}$**"** ($G_{solv}$ = −2165.902320 a.u.)

**Figure S3.** Optimized structures of $\mathbf{1_A}$, and its isomers $\mathbf{1_A}$**'** and $\mathbf{1_A}$**''**.

## 4 Solvent corrected absolute free energies (Hartree) and Cartesian coordinates (Ångström) of all structures optimized in THF.

$H_3BNH_3$

$G_{solv}$= -83.171020

| | | | |
|---|---|---|---|
| H | -1.549208 | -0.364414 | 0.007157 |
| H | -1.554175 | 1.385966 | -1.001808 |
| H | -3.200368 | 0.799086 | 0.011375 |
| H | -0.426264 | 1.554208 | 1.319953 |
| H | -1.761120 | 2.503180 | 1.314903 |
| H | -1.767404 | 1.085185 | 2.134813 |
| B | -1.981805 | 0.775040 | -0.036924 |
| N | -1.443992 | 1.535597 | 1.280151 |

$H_2NBH_2$

$G_{solv}$= -81.998026

| | | | |
|---|---|---|---|
| B | -0.554465 | 0.718255 | -0.033081 |
| H | 0.444631 | 0.508329 | -0.663493 |
| H | -1.133121 | -0.162341 | 0.541180 |
| N | -1.055612 | 2.013469 | 0.034163 |
| H | -1.891039 | 2.257271 | 0.547738 |
| H | -0.620113 | 2.801505 | -0.424679 |

acetone

$G_{solv}$= -193.057009

| | | | |
|---|---|---|---|
| C | 0.852721 | -0.089213 | 0.000154 |
| O | 1.442296 | 0.979754 | -0.002439 |
| C | -0.658685 | -0.137403 | 0.000057 |
| H | -1.032447 | 0.389882 | -0.883427 |
| H | -1.046910 | -1.158109 | 0.001727 |
| H | -1.032552 | 0.392708 | 0.881809 |
| C | 1.586953 | -1.403286 | 0.002568 |
| H | 1.293460 | -1.985517 | 0.883769 |
| H | 1.293541 | -1.988763 | -0.876505 |
| H | 2.667015 | -1.247543 | 0.002327 |

2-propanol

$G_{solv}$= -194.241437

| | | | |
|---|---|---|---|
| C | 0.868412 | -0.041773 | -0.074248 |
| O | 1.212442 | 0.485533 | -1.359114 |
| C | -0.645814 | -0.146791 | -0.034366 |
| H | -1.107221 | 0.827948 | -0.220752 |
| H | -1.001699 | -0.850880 | -0.794862 |
| H | -0.977133 | -0.501110 | 0.946939 |
| C | 1.548655 | -1.383790 | 0.168334 |
| H | 1.287199 | -1.773135 | 1.158151 |
| H | 1.235413 | -2.113711 | -0.586600 |
| H | 2.639842 | -1.287589 | 0.125657 |
| H | 1.194786 | 0.668199 | 0.700737 |
| H | 2.172393 | 0.541204 | -1.417514 |

$1_A$

$G_{solv}$= -2165.906340

| | | | |
|---|---|---|---|
| Ni | -40.589456 | 16.548823 | -31.412418 |
| C | -42.852474 | 18.611752 | -34.216356 |
| C | -41.823480 | 17.927110 | -33.601507 |
| C | -43.258183 | 17.302434 | -30.227988 |
| C | -41.953970 | 17.477176 | -32.271967 |
| C | -43.170585 | 17.764788 | -31.626661 |
| C | -44.174860 | 18.448421 | -32.283583 |
| C | -40.525230 | 17.616089 | -34.235432 |
| S | -39.410599 | 16.771094 | -33.270878 |
| S | -41.912495 | 16.450148 | -29.639232 |
| H | -42.817157 | 18.997172 | -35.228861 |
| H | -45.130595 | 18.703421 | -31.839759 |
| N | -40.247773 | 17.974684 | -35.470449 |
| Cl | -38.965758 | 15.432792 | -30.390302 |
| N | -44.314149 | 17.546062 | -29.481519 |
| C | -44.733140 | 21.179017 | -34.059008 |
| C | -45.044312 | 19.684854 | -34.206668 |
| H | -45.082986 | 19.394971 | -35.257405 |
| H | -45.997274 | 19.429863 | -33.741593 |
| N | -44.003896 | 18.853168 | -33.556603 |
| C | -44.581314 | 21.629489 | -32.639854 |
| N | -43.414125 | 21.401279 | -31.936945 |
| C | -45.488119 | 22.272807 | -31.835225 |
| C | -43.613917 | 21.906418 | -30.736262 |
| N | -44.854351 | 22.438459 | -30.629021 |
| H | -46.491136 | 22.629345 | -32.014782 |
| H | -42.903543 | 21.914571 | -29.922300 |
| H | -43.818591 | 21.412583 | -34.614773 |
| H | -45.553542 | 21.718599 | -34.540784 |
| H | -45.233016 | 22.907914 | -29.817527 |
| H | -39.346884 | 17.758195 | -35.881927 |
| H | -40.904291 | 18.465644 | -36.063520 |
| H | -44.351378 | 17.226610 | -28.520105 |
| H | -45.112165 | 18.071709 | -29.815653 |

$2_A$

$G_{solv}$= -2249.073694

| | | | |
|---|---|---|---|
| Ni | 1.269900 | -2.230052 | 0.948885 |
| C | -1.312906 | -0.127203 | -1.527707 |
| C | -0.255552 | -0.886431 | -1.067969 |

| | | | |
|---|---|---|---|
| C | -0.979256 | -1.044554 | 2.575050 |
| C | -0.137847 | -1.202876 | 0.298366 |
| C | -1.145779 | -0.708737 | 1.148607 |
| C | -2.191740 | 0.036249 | 0.644220 |
| S | 2.009951 | -2.343921 | -1.134393 |
| S | 0.383475 | -1.979245 | 2.965580 |
| H | -1.455942 | 0.165037 | -2.561023 |
| H | -3.000602 | 0.442620 | 1.238961 |
| C | -2.934042 | 2.658385 | -1.145835 |
| C | -3.346152 | 1.181579 | -1.178840 |
| H | -3.573138 | 0.862962 | -2.196894 |
| H | -4.222294 | 0.999072 | -0.554835 |
| N | -2.260976 | 0.309490 | -0.674320 |
| C | -2.557577 | 3.136881 | 0.221306 |
| N | -1.259305 | 3.033395 | 0.682132 |
| C | -3.353208 | 3.653671 | 1.212286 |
| C | -1.272107 | 3.489532 | 1.920216 |
| N | -2.518374 | 3.866194 | 2.281570 |
| H | -4.405853 | 3.890651 | 1.245178 |
| H | -0.420580 | 3.564469 | 2.580307 |
| H | -2.092309 | 2.810742 | -1.830190 |
| H | -3.782849 | 3.230028 | -1.531639 |
| H | -2.780682 | 4.274545 | 3.168852 |
| B | 1.113780 | 2.324799 | -2.387581 |
| H | 1.882000 | 2.732181 | -3.243176 |
| H | 1.581912 | 1.380760 | -1.774422 |
| H | 0.026597 | 2.035598 | -2.872677 |
| N | 0.870237 | 3.505788 | -1.334306 |
| H | 0.502647 | 4.344919 | -1.779909 |
| H | 0.199918 | 3.239633 | -0.593008 |
| H | 1.738435 | 3.775590 | -0.874740 |
| C | 0.823445 | -1.417071 | -1.924104 |
| N | 0.857426 | -1.191189 | -3.217735 |
| H | 1.622523 | -1.542500 | -3.781817 |
| H | 0.196155 | -0.584627 | -3.685956 |
| N | -1.834602 | -0.635818 | 3.488523 |
| H | -1.702118 | -0.881657 | 4.462869 |
| H | -2.661432 | -0.095312 | 3.269017 |
| Cl | 2.935431 | -3.475403 | 1.726972 |

$TS_{2,3\text{-}A}$

$G_{solv}$= -2249.053378

| | | | |
|---|---|---|---|
| Ni | -39.852169 | 16.707128 | -32.345323 |
| C | -43.240300 | 18.188624 | -34.237453 |
| C | -42.008131 | 17.643579 | -34.025939 |
| C | -41.699307 | 18.016237 | -30.320777 |
| C | -41.367359 | 17.742919 | -32.730062 |
| C | -42.248240 | 18.174860 | -31.662936 |
| C | -43.485148 | 18.692463 | -31.945212 |
| S | -39.793506 | 16.203411 | -34.503575 |
| S | -40.216632 | 17.183924 | -30.211960 |
| H | -43.748542 | 18.180445 | -35.194729 |
| H | -44.181301 | 19.052747 | -31.196652 |
| C | -45.045702 | 20.916334 | -33.771698 |
| C | -45.230551 | 19.416830 | -33.510389 |
| H | -45.659762 | 18.930915 | -34.387958 |
| H | -45.898798 | 19.243959 | -32.664798 |
| N | -43.951598 | 18.745214 | -33.218128 |
| C | -44.704178 | 21.716400 | -32.553795 |
| N | -43.442619 | 21.725219 | -31.987904 |
| C | -45.538502 | 22.511439 | -31.810239 |
| C | -43.522988 | 22.511795 | -30.929643 |
| N | -44.770499 | 23.004477 | -30.785467 |
| H | -46.580460 | 22.768271 | -31.926129 |
| H | -42.710959 | 22.755477 | -30.259899 |
| H | -44.279558 | 21.052602 | -34.543407 |
| H | -45.989120 | 21.287368 | -34.183184 |
| H | -45.076420 | 23.647415 | -30.067345 |
| B | -40.016850 | 20.046764 | -33.273092 |
| H | -38.813414 | 20.012407 | -33.212669 |
| H | -40.412660 | 18.858584 | -32.921635 |
| H | -40.518537 | 20.237074 | -34.356258 |
| N | -40.617180 | 21.010378 | -32.184656 |
| H | -40.218193 | 21.944580 | -32.288593 |
| H | -41.649423 | 21.123274 | -32.237233 |
| H | -40.381039 | 20.701865 | -31.240766 |
| C | -41.214440 | 16.966090 | -35.047133 |
| N | -41.565151 | 16.950288 | -36.321629 |
| H | -40.995147 | 16.469651 | -37.006334 |
| H | -42.378484 | 17.438744 | -36.671776 |
| N | -42.291582 | 18.495347 | -29.237306 |
| H | -41.891668 | 18.327920 | -28.322337 |
| H | -43.174978 | 18.986561 | -29.258807 |
| Cl | -37.967983 | 15.589500 | -31.904486 |

$3_A$

$G_{solv}$= -2249.057193

| | | | |
|---|---|---|---|
| Ni | 1.688770 | -1.830479 | 0.276954 |
| C | -1.455576 | -0.157164 | -1.806036 |
| C | -0.198483 | -0.613427 | -1.550285 |
| C | -0.188469 | -0.411708 | 2.198205 |
| C | 0.395750 | -0.511318 | -0.196597 |
| C | -0.613006 | -0.167249 | 0.833002 |
| C | -1.851268 | 0.271972 | 0.490302 |

S 1.915311 -2.228937 -1.890067
S 1.171415 -1.423440 2.390460
H -1.933031 -0.227596 -2.779037
H -2.620772 0.525986 1.211967
C -3.449737 2.433092 -1.452566
C -3.555502 0.930673 -1.159938
H -3.941885 0.402169 -2.034173
H -4.237720 0.749342 -0.326482
N -2.255838 0.342118 -0.817721
C -2.987733 3.236206 -0.277379
N -1.651352 3.340804 0.058222
C -3.737327 3.914805 0.648452
C -1.599519 4.069349 1.158449
N -2.838728 4.432098 1.548877
H -4.800783 4.076936 0.736138
H -0.704402 4.351233 1.692860
H -2.768511 2.586731 -2.297365
H -4.439993 2.776527 -1.766496
H -3.063794 5.008078 2.349081
B 1.665855 1.706129 -0.243618
H 1.494693 1.965601 0.910516
H 1.261356 0.311808 -0.208556
H 2.748020 1.468085 -0.692397
N 0.714598 2.433123 -1.216145
H 0.557762 1.907983 -2.079310
H -0.212105 2.694879 -0.785502
H 1.158201 3.309928 -1.504578
C 0.620843 -1.316451 -2.522009
N -0.801337 0.123241 3.244781
H -0.497884 -0.090948 4.185993
H -1.554375 0.789715 3.144836
N 0.396182 -1.258513 -3.827602
H 0.960497 -1.799191 -4.470286
H -0.325139 -0.677596 -4.231907
Cl 3.363638 -3.224240 0.830320

TS$_{3,4\text{-}A}$
G$_{solv}$= -2249.055139
Ni -40.053827 16.725098 -31.920410
C -43.250157 18.194266 -34.093470
C -41.991167 17.783725 -33.797793
C -42.149230 17.877703 -30.047034
C -41.416348 17.971939 -32.422787
C -42.513323 18.160550 -31.416878
C -43.754885 18.547524 -31.797523
S -39.792552 16.280462 -34.066106
S -40.681899 17.035760 -29.823573
H -43.705895 18.102544 -35.075703
H -44.573070 18.727963 -31.107398
C -45.107214 20.859309 -33.715887
C -45.332998 19.353665 -33.500590
H -45.713763 18.896090 -34.416610
H -46.068229 19.189866 -32.709316
N -44.095022 18.676800 -33.122943
C -44.512076 21.530972 -32.518104
N -43.145493 21.590660 -32.312564
C -45.133543 22.096080 -31.436808
C -42.949232 22.184319 -31.147285
N -44.129696 22.498814 -30.589009
H -46.176927 22.248250 -31.207522
H -41.992691 22.396625 -30.694214
H -44.449087 21.005125 -34.579606
H -46.070758 21.316099 -33.959383
H -44.253458 22.971653 -29.703130
B -40.012577 20.432725 -33.493153
H -39.506365 20.869278 -32.509673
H -40.753184 18.876000 -32.402603
H -39.559710 19.546075 -34.139952
N -41.211842 21.161174 -34.005477
H -41.578062 20.790152 -34.881555
H -42.085842 21.265105 -33.231977
H -40.969506 22.144750 -34.161959
C -41.130843 17.095840 -34.737644
N -42.881123 18.258113 -29.002842
H -42.606788 18.001549 -28.063928
H -43.723006 18.808008 -29.100072
N -41.334673 17.098384 -36.052299
H -40.729714 16.567178 -36.664598
H -42.101179 17.594541 -36.483892
Cl -38.234173 15.503444 -31.325447

4$_A$
G$_{solv}$= -2249.076062
Ni 1.549924 -1.770270 0.492222
C -1.526339 -0.097970 -1.641514
C -0.259250 -0.449595 -1.323271
C -0.607240 -0.791592 2.399070
C 0.244362 -0.423078 0.090884
C -0.908816 -0.358400 1.052782
C -2.153419 0.000475 0.655107
S 1.971655 -1.877905 -1.678646
S 0.809930 -1.723568 2.577113
H -1.926951 -0.120471 -2.651124
H -3.007153 0.057863 1.324121
C -3.513304 2.356019 -1.369114
C -3.708160 0.861158 -1.050784

| | | | |
|---|---|---|---|
| H | -4.119893 | 0.351454 | -1.925682 |
| H | -4.412735 | 0.740099 | -0.225894 |
| N | -2.450918 | 0.228744 | -0.669748 |
| C | -2.770382 | 3.070515 | -0.290281 |
| N | -1.387603 | 3.098027 | -0.243509 |
| C | -3.190527 | 3.719002 | 0.833540 |
| C | -0.975132 | 3.736307 | 0.846772 |
| N | -2.059750 | 4.117091 | 1.512913 |
| H | -4.183260 | 3.929664 | 1.197745 |
| H | 0.050644 | 3.910949 | 1.130762 |
| H | -2.967057 | 2.464357 | -2.311876 |
| H | -4.494026 | 2.818139 | -1.505441 |
| H | -2.046762 | 4.631180 | 2.386576 |
| B | 1.859895 | 2.506531 | -1.571212 |
| H | 2.251583 | 3.355487 | -0.820803 |
| H | 0.897922 | 0.458000 | 0.232637 |
| H | 2.290861 | 1.392185 | -1.550836 |
| N | 0.831746 | 2.825851 | -2.465983 |
| H | 0.529597 | 2.179522 | -3.183292 |
| H | -0.755189 | 2.730573 | -0.959351 |
| H | 0.522846 | 3.775921 | -2.627686 |
| C | 0.682996 | -0.956082 | -2.300061 |
| N | -1.360383 | -0.475681 | 3.450008 |
| H | -1.147817 | -0.846717 | 4.366398 |
| H | -2.167996 | 0.126279 | 3.371516 |
| N | 0.571360 | -0.700614 | -3.600694 |
| H | 1.213419 | -1.113354 | -4.263948 |
| H | -0.183715 | -0.142979 | -3.974373 |
| Cl | 3.296463 | -3.134290 | 1.018978 |

$5_A$

$G_{solv}$= -2167.088251

| | | | |
|---|---|---|---|
| Ni | -40.827791 | 16.224841 | -31.589103 |
| C | -42.919462 | 18.505151 | -34.300759 |
| C | -41.859136 | 17.964309 | -33.655082 |
| C | -43.492444 | 17.062939 | -30.396436 |
| C | -41.872672 | 17.725157 | -32.170342 |
| C | -43.287036 | 17.737507 | -31.659465 |
| C | -44.301390 | 18.278714 | -32.375262 |
| C | -40.676161 | 17.494059 | -34.346859 |
| S | -39.701749 | 16.393528 | -33.483863 |
| S | -42.249045 | 16.003301 | -29.909258 |
| H | -42.940794 | 18.702265 | -35.368573 |
| H | -45.332502 | 18.301192 | -32.036485 |
| N | -40.346826 | 17.894162 | -35.571501 |
| C | -44.816174 | 21.069830 | -34.237499 |
| C | -45.133382 | 19.565768 | -34.309708 |
| H | -45.205447 | 19.261022 | -35.356624 |
| H | -46.091064 | 19.356389 | -33.828665 |
| N | -44.105410 | 18.769725 | -33.647818 |
| C | -44.601394 | 21.547491 | -32.840242 |
| N | -43.405530 | 21.367083 | -32.164461 |
| C | -45.435072 | 22.158293 | -31.951248 |
| C | -43.494027 | 21.848128 | -30.928116 |
| N | -44.723165 | 22.330288 | -30.784447 |
| H | -46.457124 | 22.485935 | -32.053596 |
| H | -42.713040 | 21.848002 | -30.184114 |
| H | -43.923237 | 21.287153 | -34.833844 |
| H | -45.646936 | 21.625204 | -34.679620 |
| H | -45.073762 | 22.772452 | -29.942144 |
| H | -39.537009 | 17.509718 | -36.039662 |
| H | -40.886333 | 18.582119 | -36.077828 |
| H | -42.570082 | 20.940890 | -32.552304 |
| H | -41.287597 | 18.515046 | -31.662873 |
| Cl | -39.391989 | 14.677899 | -30.734555 |
| N | -44.567016 | 17.262105 | -29.637298 |
| H | -44.701323 | 16.734763 | -28.784949 |
| H | -45.280870 | 17.933446 | -29.883893 |

$6_{A1}$

$G_{solv}$= -2360.146540

| | | | |
|---|---|---|---|
| Ni | 0.371349 | -2.559917 | 0.669636 |
| C | -0.595839 | 0.310180 | -2.103946 |
| C | 0.168473 | -0.574131 | -1.420361 |
| C | -1.728802 | -0.680356 | 1.817611 |
| C | 0.020111 | -0.778940 | 0.057739 |
| C | -1.268045 | -0.181367 | 0.542751 |
| C | -1.991491 | 0.679899 | -0.213398 |
| C | 1.114005 | -1.455564 | -2.066551 |
| S | 1.526435 | -2.864774 | -1.194799 |
| S | -1.033446 | -2.150352 | 2.332374 |
| H | -0.517359 | 0.471214 | -3.174465 |
| H | -2.940155 | 1.105177 | 0.100949 |
| N | 1.655352 | -1.192770 | -3.252476 |
| C | -1.844329 | 3.401687 | -2.170018 |
| C | -2.401545 | 1.973119 | -2.264241 |
| H | -2.411923 | 1.659442 | -3.310164 |
| H | -3.430385 | 1.944964 | -1.897811 |
| N | -1.617081 | 1.008968 | -1.496883 |
| C | -1.842728 | 3.938632 | -0.776733 |
| N | -0.791104 | 3.741518 | 0.098990 |
| C | -2.792586 | 4.627160 | -0.079011 |
| C | -1.074473 | 4.288412 | 1.274716 |
| N | -2.288263 | 4.826768 | 1.186696 |

| | | | |
|---|---|---|---|
| H | -3.763093 | 4.991678 | -0.375349 |
| H | -0.433715 | 4.298756 | 2.141785 |
| H | -0.828309 | 3.429249 | -2.576067 |
| H | -2.464553 | 4.048811 | -2.795936 |
| H | -2.753504 | 5.325658 | 1.936119 |
| C | 2.249080 | 1.845966 | 0.056050 |
| H | 0.866838 | -0.309434 | 0.588166 |
| O | 1.487982 | 2.494479 | -0.661142 |
| H | 0.108363 | 3.261984 | -0.134005 |
| C | 2.154322 | 1.906757 | 1.553421 |
| H | 2.425760 | 0.951506 | 2.009749 |
| H | 2.880536 | 2.651743 | 1.902924 |
| H | 1.156685 | 2.212154 | 1.878482 |
| H | 1.482297 | -0.326116 | -3.742659 |
| H | 2.274119 | -1.858176 | -3.695690 |
| N | -2.621427 | -0.038766 | 2.568441 |
| H | -2.967157 | -0.455788 | 3.422327 |
| H | -2.993570 | 0.864913 | 2.314078 |
| Cl | 1.063079 | -4.535558 | 1.570333 |
| C | 3.316993 | 0.983909 | -0.536553 |
| H | 4.278705 | 1.189030 | -0.054524 |
| H | 3.075410 | -0.063794 | -0.318219 |
| H | 3.389544 | 1.129271 | -1.614912 |

$TS_{6,7\text{-}A1}$

$G_{solv}$= -2360.118141

| | | | |
|---|---|---|---|
| Ni | -41.295854 | 15.383971 | -31.507847 |
| C | -42.873514 | 17.981768 | -34.325527 |
| C | -42.007325 | 17.135995 | -33.702657 |
| C | -43.472129 | 17.095582 | -30.255275 |
| C | -42.017039 | 16.978062 | -32.241109 |
| C | -43.233139 | 17.530984 | -31.620143 |
| C | -44.061617 | 18.360956 | -32.314469 |
| C | -41.032352 | 16.303060 | -34.393161 |
| S | -40.365353 | 15.023584 | -33.482881 |
| S | -42.523494 | 15.787109 | -29.708437 |
| H | -42.891452 | 18.151057 | -35.397475 |
| H | -44.956462 | 18.809120 | -31.896001 |
| N | -40.669039 | 16.516761 | -35.647998 |
| C | -44.005466 | 21.031146 | -34.286495 |
| C | -44.671139 | 19.648698 | -34.314163 |
| H | -44.817452 | 19.326058 | -35.347294 |
| H | -45.647206 | 19.677935 | -33.825663 |
| N | -43.858202 | 18.632158 | -33.635253 |
| C | -43.823709 | 21.598032 | -32.913074 |
| N | -42.791047 | 21.215056 | -32.075214 |
| C | -44.595966 | 22.524387 | -32.263195 |
| C | -42.936849 | 21.902522 | -30.955954 |
| N | -44.017573 | 22.700321 | -31.030043 |
| H | -45.475978 | 23.068191 | -32.570358 |
| H | -42.290814 | 21.846974 | -30.092339 |
| H | -43.037129 | 20.975330 | -34.795619 |
| H | -44.637249 | 21.707080 | -34.870185 |
| H | -44.331881 | 23.336785 | -30.309473 |
| C | -40.317274 | 18.885033 | -31.717514 |
| H | -41.085035 | 17.659960 | -31.839845 |
| O | -40.752391 | 19.689601 | -32.657253 |
| H | -41.568205 | 20.265880 | -32.385802 |
| C | -40.681179 | 19.183341 | -30.290882 |
| H | -40.374172 | 18.367056 | -29.635761 |
| H | -40.146700 | 20.093572 | -29.992603 |
| H | -41.753008 | 19.362939 | -30.174512 |
| H | -40.997339 | 17.311657 | -36.179599 |
| H | -40.008663 | 15.900879 | -36.105439 |
| N | -44.357901 | 17.678092 | -29.459309 |
| H | -44.524091 | 17.319843 | -28.527741 |
| H | -44.907382 | 18.476276 | -29.747249 |
| Cl | -40.269970 | 13.621153 | -30.569449 |
| C | -38.980492 | 18.268632 | -32.005525 |
| H | -38.215868 | 19.035463 | -31.828007 |
| H | -38.786093 | 17.420853 | -31.346428 |
| H | -38.913661 | 17.953436 | -33.048912 |

$7_{A1}$

$G_{solv}$= -2360.147388

| | | | |
|---|---|---|---|
| Ni | 0.704410 | -2.311695 | 0.526555 |
| C | -0.863780 | 0.293541 | -2.296250 |
| C | -0.119928 | -0.698263 | -1.692137 |
| C | -1.428373 | -0.591139 | 1.784504 |
| C | -0.313331 | -1.013031 | -0.333360 |
| C | -1.291105 | -0.268248 | 0.352668 |
| C | -2.029808 | 0.699044 | -0.298585 |
| C | 0.934775 | -1.479989 | -2.366233 |
| S | 1.711268 | -2.654588 | -1.415243 |
| S | -0.410806 | -1.814227 | 2.374626 |
| H | -0.755592 | 0.598619 | -3.330852 |
| H | -2.813966 | 1.287688 | 0.162310 |
| N | 1.255836 | -1.279113 | -3.626383 |
| C | -1.839293 | 3.388781 | -2.175424 |
| C | -2.565967 | 2.043945 | -2.272389 |
| H | -2.689903 | 1.749549 | -3.315090 |
| H | -3.550659 | 2.089676 | -1.805706 |
| N | -1.810832 | 0.958206 | -1.602994 |
| C | -1.724746 | 3.924480 | -0.783551 |
| N | -0.820664 | 3.408722 | 0.124331 |
| C | -2.442127 | 4.930915 | -0.189411 |

| | | | |
|---|---|---|---|
| C | -0.984360 | 4.098452 | 1.236674 |
| N | -1.957855 | 5.022948 | 1.092318 |
| H | -3.221571 | 5.578698 | -0.560623 |
| H | -0.424474 | 3.961709 | 2.150421 |
| H | -0.844300 | 3.291735 | -2.623516 |
| H | -2.404722 | 4.094017 | -2.791178 |
| H | -2.251367 | 5.690409 | 1.793024 |
| C | 2.090159 | 1.176247 | 0.452721 |
| H | 1.772574 | 0.124668 | 0.366502 |
| O | 1.500510 | 1.920553 | -0.606561 |
| H | 0.688567 | 2.369752 | -0.295702 |
| C | 1.645547 | 1.678557 | 1.820528 |
| H | 2.096050 | 1.069932 | 2.611152 |
| H | 1.948494 | 2.721781 | 1.969403 |
| H | 0.556598 | 1.612155 | 1.927237 |
| H | 0.817151 | -0.573027 | -4.203853 |
| H | 1.991929 | -1.823137 | -4.062119 |
| N | -2.278617 | 0.049491 | 2.558870 |
| H | -2.360312 | -0.187320 | 3.541017 |
| H | -2.887026 | 0.781453 | 2.215835 |
| Cl | 1.937988 | -3.840538 | 1.559820 |
| C | 3.599860 | 1.213829 | 0.281857 |
| H | 3.978024 | 2.235341 | 0.404599 |
| H | 4.085612 | 0.567832 | 1.020758 |
| H | 3.879358 | 0.859224 | -0.715611 |

N-methylacetonimine
$G_{solv}$= -212.439490

| | | | |
|---|---|---|---|
| C | 0.780508 | 1.317875 | 0.018091 |
| N | 1.394099 | 2.429147 | 0.175374 |
| C | 1.467510 | -0.010774 | -0.188251 |
| H | 0.745424 | -0.825828 | -0.272024 |
| H | 2.074895 | 0.007940 | -1.101065 |
| H | 2.145985 | -0.230858 | 0.644079 |
| C | -0.721839 | 1.306779 | 0.029775 |
| H | -1.104241 | 0.906784 | -0.917086 |
| H | -1.091177 | 0.648537 | 0.825293 |
| H | -1.118308 | 2.313175 | 0.181162 |
| C | 2.849695 | 2.424567 | 0.158479 |
| H | 3.213479 | 3.447007 | 0.285684 |
| H | 3.267749 | 1.812952 | 0.969950 |
| H | 3.250717 | 2.036545 | -0.787993 |

N-methylisopropylamine
$G_{solv}$= -213.636037

| | | | |
|---|---|---|---|
| C | 0.654567 | 0.990043 | -0.395325 |
| N | 1.354746 | 2.087557 | 0.279518 |
| C | 1.233088 | -0.361131 | 0.021271 |
| H | 0.673349 | -1.175127 | -0.451548 |
| H | 2.280921 | -0.468505 | -0.276166 |
| H | 1.171041 | -0.483183 | 1.109089 |
| C | -0.832453 | 1.074842 | -0.064852 |
| H | -1.389235 | 0.286734 | -0.581698 |
| H | -0.994078 | 0.961293 | 1.013429 |
| H | -1.252126 | 2.039259 | -0.374303 |
| C | 2.732990 | 2.282234 | -0.153748 |
| H | 3.106847 | 3.228248 | 0.248097 |
| H | 3.375939 | 1.485372 | 0.232668 |
| H | 2.845356 | 2.304977 | -1.252052 |
| H | 0.770340 | 1.083220 | -1.492226 |
| H | 0.839423 | 2.945450 | 0.105018 |

$TS_{6,7\text{-}A2}$
$G_{solv}$= -2379.539020

| | | | |
|---|---|---|---|
| Ni | 0.581821 | -2.703563 | 0.439999 |
| C | -0.890887 | 0.197478 | -2.047494 |
| C | 0.017643 | -0.657923 | -1.521065 |
| C | -1.278966 | -0.864029 | 1.994496 |
| C | 0.119532 | -0.911332 | -0.049071 |
| C | -1.069968 | -0.344118 | 0.663463 |
| C | -1.948397 | 0.494039 | 0.060245 |
| C | 0.872850 | -1.490023 | -2.337132 |
| S | 1.433778 | -2.920236 | -1.594304 |
| S | -0.514973 | -2.351615 | 2.334224 |
| H | -0.997962 | 0.371303 | -3.114040 |
| H | -2.837904 | 0.877252 | 0.551042 |
| N | 1.223373 | -1.168222 | -3.579410 |
| C | -2.353563 | 3.145183 | -2.072158 |
| C | -2.823779 | 1.694193 | -1.913593 |
| H | -3.041916 | 1.279595 | -2.901028 |
| H | -3.746147 | 1.655016 | -1.329652 |
| N | -1.830556 | 0.836549 | -1.268052 |
| C | -2.240151 | 3.893214 | -0.784683 |
| N | -1.172126 | 3.761821 | 0.083627 |
| C | -3.114229 | 4.777824 | -0.217732 |
| C | -1.384346 | 4.545355 | 1.131953 |
| N | -2.556076 | 5.167337 | 0.977112 |
| H | -4.061617 | 5.160577 | -0.563021 |
| H | -0.720333 | 4.664685 | 1.973953 |
| H | -1.395549 | 3.160999 | -2.602036 |
| H | -3.079416 | 3.662620 | -2.705850 |
| H | -2.954601 | 5.832822 | 1.627661 |
| C | 1.760147 | 1.951219 | 0.670061 |
| H | 1.044938 | -0.455669 | 0.341013 |

| | | | |
|---|---|---|---|
| H | -0.183623 | 3.141575 | -0.055002 |
| C | 1.196298 | 1.824301 | 2.050855 |
| H | 1.206637 | 0.771466 | 2.353906 |
| H | 1.839543 | 2.361193 | 2.757431 |
| H | 0.177666 | 2.208030 | 2.123155 |
| H | 0.958285 | -0.290180 | -4.002570 |
| H | 1.777381 | -1.802595 | -4.139123 |
| N | -1.997912 | -0.226156 | 2.914931 |
| H | -2.172568 | -0.649800 | 3.816194 |
| H | -2.387192 | 0.691394 | 2.750362 |
| Cl | 1.417193 | -4.704745 | 1.140052 |
| C | 3.136199 | 1.401958 | 0.435361 |
| H | 3.512426 | 0.890295 | 1.322275 |
| H | 3.139638 | 0.697807 | -0.403742 |
| H | 3.825211 | 2.215304 | 0.178102 |
| N | 1.070191 | 2.517500 | -0.252397 |
| C | 1.607924 | 2.656798 | -1.598195 |
| H | 0.877415 | 3.187736 | -2.213310 |
| H | 2.542253 | 3.228576 | -1.601211 |
| H | 1.800417 | 1.678273 | -2.052287 |

$6_{A2}$

$G_{solv}$= -2379.539034

| | | | |
|---|---|---|---|
| Ni | 0.727492 | -2.649799 | 0.442483 |
| C | -0.744860 | 0.251313 | -2.045147 |
| C | 0.163592 | -0.604131 | -1.518650 |
| C | -1.133280 | -0.810142 | 1.996827 |
| C | 0.265361 | -0.857542 | -0.046650 |
| C | -0.924165 | -0.290271 | 0.665799 |
| C | -1.802533 | 0.547896 | 0.062510 |
| C | 1.018797 | -1.436305 | -2.334647 |
| S | 1.579572 | -2.866555 | -1.591765 |
| S | -0.369394 | -2.297760 | 2.336628 |
| H | -0.851858 | 0.425129 | -3.111702 |
| H | -2.692066 | 0.931130 | 0.553239 |
| N | 1.369419 | -1.114552 | -3.576910 |
| C | -2.207601 | 3.199030 | -2.069791 |
| C | -2.677779 | 1.748019 | -1.911390 |
| H | -2.895808 | 1.333463 | -2.898866 |
| H | -3.600199 | 1.708778 | -1.327535 |
| N | -1.684585 | 0.890395 | -1.265778 |
| C | -2.094280 | 3.946891 | -0.782210 |
| N | -1.026360 | 3.815261 | 0.086188 |
| C | -2.968339 | 4.831498 | -0.215223 |
| C | -1.238611 | 4.598672 | 1.134599 |
| N | -2.410267 | 5.220792 | 0.979731 |
| H | -3.915658 | 5.214387 | -0.560551 |
| H | -0.574674 | 4.717820 | 1.976686 |
| H | -1.249550 | 3.214908 | -2.599602 |
| H | -2.933421 | 3.716524 | -2.703475 |
| H | -2.808791 | 5.886228 | 1.630333 |
| C | 1.906340 | 2.005026 | 0.672421 |
| H | 1.190765 | -0.401925 | 0.343491 |
| H | -0.037835 | 3.194902 | -0.052486 |
| C | 1.342729 | 1.878187 | 2.053319 |
| H | 1.353249 | 0.825388 | 2.356490 |
| H | 1.986026 | 2.415239 | 2.759727 |
| H | 0.324066 | 2.261809 | 2.125749 |
| H | 1.104417 | -0.236503 | -4.000108 |
| H | 1.923409 | -1.748979 | -4.136579 |
| N | -1.852258 | -0.172219 | 2.917202 |
| H | -2.027001 | -0.595845 | 3.818457 |
| H | -2.241479 | 0.745348 | 2.752593 |
| Cl | 1.562655 | -4.651037 | 1.142627 |
| C | 3.282442 | 1.455959 | 0.437568 |
| H | 3.658848 | 0.944373 | 1.324451 |
| H | 3.285883 | 0.751788 | -0.401518 |
| H | 3.971307 | 2.269398 | 0.180210 |
| N | 1.216145 | 2.571099 | -0.249989 |
| C | 1.753616 | 2.710407 | -1.595890 |
| H | 1.022912 | 3.241205 | -2.210892 |
| H | 2.687848 | 3.282341 | -1.599088 |
| H | 1.946202 | 1.731893 | -2.049969 |

$7_{A2}$

$G_{solv}$= -2379.545281

| | | | |
|---|---|---|---|
| Ni | 0.728193 | -2.605775 | 0.468879 |
| C | -0.760508 | 0.242986 | -2.075440 |
| C | 0.149933 | -0.605145 | -1.536690 |
| C | -1.136422 | -0.737759 | 1.986424 |
| C | 0.258394 | -0.827353 | -0.059351 |
| C | -0.925917 | -0.241946 | 0.647309 |
| C | -1.801793 | 0.589568 | 0.029910 |
| C | 0.997248 | -1.457241 | -2.339272 |
| S | 1.573291 | -2.864051 | -1.563235 |
| S | -0.368644 | -2.217791 | 2.355570 |
| H | -0.874114 | 0.394636 | -3.144597 |
| H | -2.684518 | 0.991688 | 0.516997 |
| N | 1.334266 | -1.170746 | -3.594500 |
| C | -2.205005 | 3.234414 | -2.046272 |
| C | -2.671040 | 1.776563 | -1.950959 |
| H | -2.857293 | 1.387129 | -2.954950 |
| H | -3.608398 | 1.712272 | -1.393101 |
| N | -1.691754 | 0.900486 | -1.304900 |
| C | -2.140452 | 3.945504 | -0.731649 |
| N | -1.103595 | 3.767244 | 0.164401 |

C -3.047513 4.822959 -0.195933
C -1.379293 4.526678 1.209671
N -2.546945 5.176251 1.032872
H -3.975307 5.221012 -0.577287
H -0.769656 4.632827 2.095117
H -1.226855 3.263045 -2.539891
H -2.908405 3.761321 -2.698495
H -2.968113 5.827049 1.682174
C 2.006740 1.953534 0.683794
H 1.189142 -0.369097 0.318270
H 0.399179 2.913733 -0.079483
C 1.409412 1.898026 2.044255
H 1.370786 0.852970 2.369892
H 2.074659 2.419800 2.740956
H 0.411809 2.336132 2.084815
H 1.048787 -0.313108 -4.045646
H 1.874450 -1.825060 -4.144858
N -1.859290 -0.086299 2.894256
H -2.037565 -0.496752 3.800906
H -2.254788 0.825546 2.712174
Cl 1.578420 -4.583588 1.212826
C 3.368046 1.395648 0.458798
H 3.712332 0.851458 1.337982
H 3.384979 0.728662 -0.408828
H 4.065325 2.217283 0.253524
N 1.346976 2.499173 -0.280633
C 1.837419 2.642640 -1.644930
H 1.084314 3.183922 -2.217624
H 2.773272 3.206580 -1.655901
H 2.002109 1.660258 -2.095240

$TS_{7,8\text{-}A2}$
$G_{solv}$= -2379.516261
Ni -41.084628 15.484692 -31.721368
C -42.952796 18.189592 -34.256293
C -42.020584 17.333078 -33.737162
C -43.284726 16.969877 -30.246384
C -42.005239 17.003785 -32.330488
C -43.156594 17.496223 -31.601230
C -44.053372 18.353021 -32.179309
C -40.992597 16.652449 -34.523285
S -40.181184 15.374996 -33.743533
S -42.247979 15.678722 -29.845171
H -43.006166 18.461854 -35.305332
H -44.922050 18.749410 -31.664815
N -40.688968 17.013274 -35.757970
C -44.395473 21.051738 -34.231131
C -44.926686 19.620349 -34.107358
H -45.166107 19.221329 -35.095737
H -45.835262 19.592582 -33.503495
N -43.950323 18.703636 -33.489137
C -44.140384 21.726566 -32.920736
N -43.013029 21.461548 -32.169201
C -44.921974 22.643290 -32.264298
C -43.113540 22.208425 -31.086638
N -44.252673 22.934227 -31.101141
H -45.861567 23.108227 -32.521523
H -42.396018 22.252624 -30.279942
H -43.477490 21.044695 -34.829615
H -45.142495 21.619213 -34.794281
H -44.545023 23.595125 -30.393973
C -40.270566 18.892279 -31.677371
H -40.936954 17.837502 -31.881779
H -41.390567 20.377159 -32.553410
C -40.805180 19.307954 -30.318604
H -40.693654 18.497038 -29.594785
H -40.240691 20.175772 -29.960298
H -41.860933 19.588757 -30.376601
H -41.123060 17.801217 -36.219570
H -39.981203 16.514090 -36.282582
N -44.147874 17.462518 -29.374479
H -44.231948 17.063452 -28.447836
H -44.748575 18.246742 -29.591426
Cl -39.879641 13.765457 -30.951360
C -38.852229 18.353812 -31.655028
H -38.157316 19.161848 -31.396660
H -38.765518 17.565866 -30.902950
H -38.554539 17.936313 -32.619206
N -40.565165 19.788189 -32.682641
C -39.756722 20.015286 -33.860592
H -40.307908 20.681259 -34.528921
H -38.798973 20.493520 -33.618863
H -39.539735 19.090993 -34.407903

$8_{A2}$
$G_{solv}$= -2379.531755
Ni 0.639981 -2.530419 0.483511
C -0.950724 0.228911 -2.175719
C -0.205952 -0.798242 -1.633649
C -1.424476 -0.829669 1.873326
C -0.362760 -1.166278 -0.285280
C -1.310122 -0.439376 0.456719
C -2.041954 0.573779 -0.127564
C 0.817090 -1.563342 -2.368437

| | | | |
|---|---|---|---|
| S | 1.617081 | -2.776667 | -1.488673 |
| S | -0.454960 | -2.132627 | 2.368851 |
| H | -0.870518 | 0.566743 | -3.202919 |
| H | -2.799530 | 1.154514 | 0.383071 |
| N | 1.099391 | -1.310879 | -3.628776 |
| C | -1.976303 | 3.361882 | -1.834914 |
| C | -2.637142 | 1.992572 | -2.032867 |
| H | -2.725537 | 1.765276 | -3.095782 |
| H | -3.635624 | 1.965921 | -1.593438 |
| N | -1.858378 | 0.885995 | -1.425714 |
| C | -2.072201 | 3.918580 | -0.447432 |
| N | -1.236626 | 3.512985 | 0.573875 |
| C | -2.957949 | 4.855315 | 0.024939 |
| C | -1.612782 | 4.194115 | 1.637888 |
| N | -2.651113 | 5.014431 | 1.352911 |
| H | -3.747728 | 5.412030 | -0.456543 |
| H | -1.164206 | 4.139063 | 2.619212 |
| H | -0.928311 | 3.303284 | -2.149300 |
| H | -2.480675 | 4.044249 | -2.525769 |
| H | -3.098208 | 5.655240 | 1.994423 |
| C | 2.154767 | 1.153977 | 0.553123 |
| H | 2.185314 | 0.072114 | 0.342888 |
| H | 0.902480 | 2.632925 | -0.141810 |
| C | 1.498014 | 1.359561 | 1.914981 |
| H | 1.982167 | 0.741261 | 2.676700 |
| H | 1.581119 | 2.408999 | 2.224837 |
| H | 0.435542 | 1.105071 | 1.887551 |
| H | 0.640919 | -0.580306 | -4.157403 |
| H | 1.813226 | -1.840600 | -4.115774 |
| N | -2.214100 | -0.182074 | 2.703407 |
| H | -2.271148 | -0.451952 | 3.678646 |
| H | -2.772723 | 0.613038 | 2.419292 |
| Cl | 1.847698 | -4.152215 | 1.402737 |
| C | 3.601656 | 1.661804 | 0.591944 |
| H | 3.626762 | 2.747271 | 0.751524 |
| H | 4.144731 | 1.183644 | 1.414432 |
| H | 4.143620 | 1.437623 | -0.332083 |
| N | 1.309954 | 1.763475 | -0.476223 |
| C | 1.914576 | 1.928499 | -1.784089 |
| H | 1.156483 | 2.284768 | -2.491535 |
| H | 2.759270 | 2.634415 | -1.821560 |
| H | 2.280253 | 0.960165 | -2.148635 |

acetophenone

$G_{solv}$= -384.690957

| | | | |
|---|---|---|---|
| C | -1.188034 | 1.027585 | 0.053061 |
| C | 0.192828 | 1.016518 | 0.239332 |
| C | 0.913193 | 2.217431 | 0.254504 |
| C | 0.229796 | 3.427934 | 0.080813 |
| C | -1.148317 | 3.438547 | -0.104475 |
| C | -1.859555 | 2.237108 | -0.119080 |
| H | -1.738580 | 0.091851 | 0.041676 |
| H | 0.700809 | 0.066525 | 0.370831 |
| H | 0.792519 | 4.355887 | 0.093843 |
| H | -1.669790 | 4.381572 | -0.237987 |
| H | -2.935736 | 2.244603 | -0.265159 |
| C | 2.400254 | 2.248384 | 0.449208 |
| O | 3.001327 | 3.313155 | 0.449824 |
| C | 3.135346 | 0.946574 | 0.643050 |
| H | 2.759499 | 0.420909 | 1.527340 |
| H | 2.987284 | 0.287827 | -0.219078 |
| H | 4.200031 | 1.148360 | 0.766644 |

1-phenyl-ethanol

$G_{solv}$= -385.874726

| | | | |
|---|---|---|---|
| C | -38.787143 | 20.652993 | -34.883916 |
| C | -38.103556 | 19.504110 | -35.284222 |
| C | -38.123156 | 18.371770 | -34.470470 |
| C | -38.824615 | 18.389994 | -33.265010 |
| C | -39.507835 | 19.538033 | -32.855200 |
| C | -39.482528 | 20.670241 | -33.675911 |
| H | -38.777475 | 21.538403 | -35.513315 |
| H | -37.562322 | 19.490995 | -36.225780 |
| H | -37.597818 | 17.471511 | -34.776414 |
| H | -38.842455 | 17.501950 | -32.636859 |
| H | -40.014373 | 21.565802 | -33.366707 |
| C | -40.225503 | 19.561543 | -31.515905 |
| H | -40.445496 | 18.523852 | -31.226729 |
| O | -41.439763 | 20.306059 | -31.569480 |
| H | -42.006594 | 19.918465 | -32.245845 |
| C | -39.368266 | 20.198843 | -30.430963 |
| H | -38.428941 | 19.649442 | -30.318095 |
| H | -39.134138 | 21.236703 | -30.690815 |
| H | -39.898567 | 20.186138 | -29.473853 |

$6_{A3}$

$G_{solv}$= -2551.780704

| | | | |
|---|---|---|---|
| Ni | -0.030985 | -2.633583 | 0.560263 |
| C | -1.120484 | 0.331548 | -2.061742 |
| C | -0.322619 | -0.573233 | -1.448274 |
| C | -2.034239 | -0.761397 | 1.888998 |
| C | -0.400716 | -0.834042 | 0.023943 |
| C | -1.641310 | -0.226107 | 0.606080 |
| C | -2.398615 | 0.664542 | -0.082137 |
| C | 0.582455 | -1.440609 | -2.171641 |
| S | 1.025734 | -2.877511 | -1.369242 |

| | | | |
|---|---|---|---|
| S | -1.337235 | -2.261614 | 2.308862 |
| H | -1.105977 | 0.517492 | -3.131058 |
| H | -3.320808 | 1.089901 | 0.301300 |
| N | 1.052480 | -1.149879 | -3.381228 |
| C | -2.411787 | 3.369590 | -2.177004 |
| C | -2.970851 | 1.941775 | -2.109021 |
| H | -3.096952 | 1.563412 | -3.126227 |
| H | -3.952747 | 1.942151 | -1.630502 |
| N | -2.104505 | 1.018609 | -1.378477 |
| C | -2.368289 | 4.063495 | -0.856408 |
| N | -1.343106 | 3.900428 | 0.057595 |
| C | -3.258020 | 4.921358 | -0.277252 |
| C | -1.584536 | 4.631960 | 1.139800 |
| N | -2.746288 | 5.253321 | 0.956818 |
| H | -4.190722 | 5.322704 | -0.639823 |
| H | -0.949041 | 4.708067 | 2.007648 |
| H | -1.411659 | 3.352853 | -2.621733 |
| H | -3.054321 | 3.949138 | -2.845287 |
| H | -3.172340 | 5.889071 | 1.620693 |
| C | 4.022258 | 0.122007 | -1.815745 |
| C | 4.693219 | -0.731005 | -0.937926 |
| C | 4.360453 | -0.746035 | 0.416290 |
| C | 3.355280 | 0.087494 | 0.895021 |
| C | 2.671200 | 0.940378 | 0.017867 |
| C | 3.014175 | 0.951586 | -1.340972 |
| H | 4.281921 | 0.132302 | -2.869763 |
| H | 5.473230 | -1.388316 | -1.310016 |
| H | 4.875964 | -1.415205 | 1.097420 |
| H | 3.101618 | 0.056029 | 1.949171 |
| H | 2.481148 | 1.613222 | -2.015564 |
| C | 1.558331 | 1.806694 | 0.483059 |
| H | 0.490030 | -0.418547 | 0.519737 |
| O | 0.945734 | 2.501707 | -0.332517 |
| H | -0.474285 | 3.335948 | -0.076964 |
| C | 1.192756 | 1.843326 | 1.941294 |
| H | 1.016009 | 0.839917 | 2.339077 |
| H | 2.018745 | 2.280543 | 2.513408 |
| H | 0.297596 | 2.449634 | 2.086570 |
| H | 0.889550 | -0.253278 | -3.817108 |
| H | 1.666084 | -1.794429 | -3.860963 |
| N | -2.871495 | -0.136738 | 2.715104 |
| H | -3.171069 | -0.579304 | 3.573618 |
| H | -3.243390 | 0.780915 | 2.515012 |
| Cl | 0.697015 | -4.638373 | 1.363553 |

TS$_{6,7-A3}$

G$_{solv}$= -2551.752487

| | | | |
|---|---|---|---|
| Ni | -41.298237 | 15.388951 | -31.489351 |
| C | -42.836005 | 17.995784 | -34.317922 |
| C | -41.976963 | 17.148246 | -33.684118 |
| C | -43.469013 | 17.115750 | -30.248868 |
| C | -42.018128 | 16.979362 | -32.230059 |
| C | -43.229740 | 17.537945 | -31.619988 |
| C | -44.052273 | 18.368266 | -32.322991 |
| C | -40.986013 | 16.318447 | -34.361406 |
| S | -40.356466 | 15.019467 | -33.457604 |
| S | -42.518852 | 15.815132 | -29.687794 |
| H | -42.835062 | 18.170828 | -35.389128 |
| H | -44.948891 | 18.820495 | -31.913133 |
| N | -40.597273 | 16.545341 | -35.606038 |
| C | -43.982957 | 21.041500 | -34.273953 |
| C | -44.640320 | 19.655692 | -34.326287 |
| H | -44.764884 | 19.340655 | -35.364403 |
| H | -45.625246 | 19.674727 | -33.855515 |
| N | -43.834392 | 18.636733 | -33.640860 |
| C | -43.840309 | 21.605332 | -32.894464 |
| N | -42.838660 | 21.212623 | -32.024280 |
| C | -44.625528 | 22.538296 | -32.269802 |
| C | -43.015643 | 21.899955 | -30.909823 |
| N | -44.086487 | 22.708257 | -31.018293 |
| H | -45.490585 | 23.089484 | -32.605103 |
| H | -42.396798 | 21.840742 | -30.026862 |
| H | -43.001755 | 20.993497 | -34.758821 |
| H | -44.603794 | 21.715781 | -34.871131 |
| H | -44.418075 | 23.348039 | -30.308591 |
| C | -37.319495 | 17.795879 | -33.736371 |
| C | -36.691138 | 16.908674 | -32.863849 |
| C | -37.224319 | 16.686827 | -31.593162 |
| C | -38.383879 | 17.341742 | -31.198878 |
| C | -39.021290 | 18.233636 | -32.072302 |
| C | -38.481783 | 18.453797 | -33.345652 |
| H | -36.905647 | 17.973234 | -34.724411 |
| H | -35.789564 | 16.387877 | -33.171716 |
| H | -36.745576 | 15.988632 | -30.914197 |
| H | -38.801125 | 17.128583 | -30.220343 |
| H | -38.974039 | 19.141684 | -34.023740 |
| C | -40.312889 | 18.871194 | -31.705427 |
| H | -41.073904 | 17.678793 | -31.810297 |
| O | -40.785133 | 19.694278 | -32.625131 |
| H | -41.589842 | 20.256325 | -32.325239 |
| C | -40.641378 | 19.192671 | -30.271505 |
| H | -40.361691 | 18.389021 | -29.591494 |
| H | -40.093172 | 20.099107 | -29.990564 |
| H | -41.711389 | 19.386213 | -30.159777 |

| | | | |
|---|---|---|---|
| H | -40.904302 | 17.353405 | -36.130150 |
| H | -39.917409 | 15.941311 | -36.049886 |
| N | -44.357201 | 17.704656 | -29.461690 |
| H | -44.521478 | 17.358529 | -28.525008 |
| H | -44.906597 | 18.499889 | -29.758276 |
| Cl | -40.312399 | 13.606316 | -30.551447 |

$7_{A3}$

$G_{solv}$= -2551.782248

| | | | |
|---|---|---|---|
| Ni | 0.264207 | -2.469486 | 0.445166 |
| C | -1.114701 | 0.364323 | -2.249767 |
| C | -0.387978 | -0.654401 | -1.670306 |
| C | -1.737638 | -0.656079 | 1.789417 |
| C | -0.615163 | -1.027715 | -0.332656 |
| C | -1.577079 | -0.281254 | 0.372736 |
| C | -2.305234 | 0.708788 | -0.256242 |
| C | 0.633114 | -1.460404 | -2.368067 |
| S | 1.213246 | -2.811750 | -1.525385 |
| S | -0.816474 | -1.980722 | 2.318001 |
| H | -0.997483 | 0.693324 | -3.275686 |
| H | -3.099560 | 1.275156 | 0.214276 |
| N | 1.058707 | -1.152654 | -3.575818 |
| C | -2.278821 | 3.467745 | -2.011144 |
| C | -2.869383 | 2.067872 | -2.212872 |
| H | -2.897177 | 1.821830 | -3.274575 |
| H | -3.886054 | 2.004090 | -1.821389 |
| N | -2.075458 | 1.004805 | -1.550358 |
| C | -2.428122 | 4.025705 | -0.629481 |
| N | -1.589609 | 3.660743 | 0.405746 |
| C | -3.354670 | 4.924079 | -0.166119 |
| C | -2.001339 | 4.327809 | 1.466861 |
| N | -3.067051 | 5.099558 | 1.164774 |
| H | -4.161195 | 5.446914 | -0.657103 |
| H | -1.554792 | 4.295973 | 2.449681 |
| H | -1.222636 | 3.457435 | -2.301208 |
| H | -2.799260 | 4.122782 | -2.715857 |
| H | -3.544097 | 5.725637 | 1.799742 |
| C | 3.908513 | -0.074020 | -1.565440 |
| C | 4.434496 | -1.048822 | -0.719544 |
| C | 3.940431 | -1.171803 | 0.579118 |
| C | 2.931533 | -0.321586 | 1.024769 |
| C | 2.406584 | 0.666023 | 0.185641 |
| C | 2.899934 | 0.778508 | -1.115663 |
| H | 4.282870 | 0.022731 | -2.581176 |
| H | 5.215115 | -1.716855 | -1.071423 |
| H | 4.327578 | -1.941513 | 1.240130 |
| H | 2.544550 | -0.429706 | 2.035571 |
| H | 2.488256 | 1.536692 | -1.774509 |
| C | 1.363215 | 1.626427 | 0.716720 |
| H | 0.692442 | 1.059363 | 1.383093 |
| O | 0.624161 | 2.153306 | -0.367931 |
| H | -0.093347 | 2.719679 | -0.009590 |
| C | 2.013363 | 2.743740 | 1.536357 |
| H | 2.568510 | 2.331043 | 2.385028 |
| H | 2.706338 | 3.317402 | 0.911777 |
| H | 1.249421 | 3.425549 | 1.925908 |
| H | 0.752294 | -0.328176 | -4.075601 |
| H | 1.769600 | -1.716923 | -4.025980 |
| N | -2.537687 | 0.011148 | 2.594775 |
| H | -2.633944 | -0.258433 | 3.567282 |
| H | -3.087295 | 0.802950 | 2.286534 |
| Cl | 1.262727 | -4.231943 | 1.363575 |

3-methyl-2-butanone

$G_{solv}$= -271.617237

| | | | |
|---|---|---|---|
| C | -40.286353 | 19.228918 | -31.714606 |
| O | -41.111469 | 19.102945 | -32.604823 |
| C | -40.719060 | 19.525909 | -30.297701 |
| H | -40.832137 | 18.577797 | -29.758360 |
| H | -39.980345 | 20.126756 | -29.760155 |
| H | -41.686166 | 20.032677 | -30.302739 |
| C | -38.476025 | 18.835724 | -33.440948 |
| H | -38.892425 | 17.882399 | -33.782927 |
| H | -38.887719 | 19.626173 | -34.074721 |
| H | -37.391663 | 18.806958 | -33.590117 |
| C | -38.213333 | 17.965612 | -31.075021 |
| H | -38.682395 | 17.000860 | -31.299955 |
| H | -37.138727 | 17.867580 | -31.257088 |
| H | -38.353126 | 18.175232 | -30.010472 |
| C | -38.793066 | 19.075303 | -31.969229 |
| H | -38.341622 | 20.026593 | -31.651277 |

3-methyl-2-butanol

$G_{solv}$= -272.800312

| | | | |
|---|---|---|---|
| C | -40.333155 | 19.153540 | -31.728437 |
| H | -40.795447 | 18.205512 | -32.050953 |
| O | -40.802298 | 20.209698 | -32.572012 |
| H | -41.754147 | 20.291565 | -32.452801 |
| C | -40.723321 | 19.424368 | -30.279404 |
| H | -40.488775 | 18.571944 | -29.635140 |
| H | -40.201529 | 20.308989 | -29.896717 |
| H | -41.802388 | 19.602077 | -30.202699 |
| C | -38.504171 | 18.749719 | -33.422591 |
| H | -38.906872 | 17.771698 | -33.716905 |
| H | -38.934755 | 19.505796 | -34.084174 |
| H | -37.421240 | 18.727379 | -33.587572 |

C -38.190890 17.978570 -31.050564
H -38.680283 17.005885 -31.189605
H -37.130809 17.852492 -31.295056
H -38.253962 18.240510 -29.990109
C -38.820152 19.043554 -31.953525
H -38.389051 20.020899 -31.693111

$6_{A4}$
$G_{solv}$= -2438.705677
Ni -41.282959 15.382078 -31.635685
C -42.983158 18.046972 -34.326675
C -42.190378 17.061196 -33.774135
C -43.392824 17.035248 -30.257519
C -42.343764 16.691942 -32.423737
C -43.320641 17.387816 -31.686797
C -44.116822 18.338875 -32.290898
C -41.120306 16.338314 -34.489014
S -40.355815 15.091578 -33.622178
S -42.328930 15.821319 -29.733237
H -42.914129 18.390389 -35.352413
H -44.915133 18.874658 -31.792704
N -40.762113 16.644474 -35.717302
C -44.166966 21.094828 -34.058382
C -44.782062 19.700146 -34.212852
H -44.884315 19.445201 -35.268062
H -45.767807 19.644999 -33.748244
N -43.945475 18.643857 -33.592919
C -44.116716 21.601142 -32.650896
N -43.188015 21.135796 -31.740672
C -44.918967 22.533332 -32.044208
C -43.422614 21.779332 -30.613646
N -44.462463 22.629870 -30.752858
H -45.741589 23.127483 -32.411986
H -42.867232 21.665585 -29.694046
H -43.161297 21.093127 -34.492932
H -44.779194 21.770194 -34.662884
H -44.819250 23.252312 -30.040326
C -40.131246 19.044982 -31.730848
H -40.376249 17.974266 -31.840365
O -40.881496 19.785508 -32.684894
H -41.656906 20.204751 -32.260247
C -40.496536 19.455674 -30.309517
H -40.011218 18.808107 -29.574524
H -40.200743 20.493236 -30.112957
H -41.578609 19.367914 -30.157139
H -41.174229 17.403837 -36.243776
H -40.004565 16.144498 -36.168712
N -44.218543 17.649946 -29.436447
H -44.250729 17.401353 -28.454618
H -44.858193 18.372870 -29.740649
Cl -39.996281 13.838807 -30.691328
C -38.370700 18.786013 -33.514413
H -38.574560 17.717505 -33.662732
H -38.996492 19.355670 -34.206715
H -37.321137 18.960866 -33.775605
C -37.776082 18.344973 -31.115443
H -38.108783 17.298556 -31.112925
H -36.729563 18.358726 -31.438289
H -37.806881 18.711555 -30.084920
C -38.639133 19.185406 -32.060678
H -38.376982 20.246114 -31.936767

$TS_{6,7\text{-}A4}$
$G_{solv}$= -2438.675874
Ni -40.899819 15.567885 -31.660230
C -42.880555 18.100684 -34.259939
C -41.894807 17.325799 -33.727946
C -43.082265 17.071644 -30.191061
C -41.764072 17.142167 -32.276433
C -42.975020 17.567170 -31.553605
C -43.932323 18.328027 -32.157691
C -40.955468 16.554165 -34.528861
S -40.160686 15.276983 -33.727058
S -41.984920 15.843416 -29.750979
H -43.002453 18.276538 -35.323983
H -44.829968 18.675352 -31.657622
N -40.711787 16.826386 -35.800593
C -44.321858 21.001113 -34.139669
C -44.839166 19.558690 -34.088691
H -45.046634 19.201634 -35.099913
H -45.764078 19.496439 -33.511949
N -43.863331 18.643137 -33.482038
C -44.123419 21.626870 -32.795158
N -43.040883 21.332307 -31.986139
C -44.924622 22.529804 -32.147435
C -43.185885 22.048282 -30.885133
N -44.313870 22.779768 -30.942853
H -45.845782 23.010886 -32.438186
H -42.506219 22.059125 -30.045870
H -43.384157 21.025081 -34.706000
H -45.053588 21.589369 -34.701134
H -44.639674 23.420688 -30.231525
C -40.310353 19.249308 -31.717849
H -40.842224 17.913460 -31.928833

O -40.878915 20.003655 -32.634130
H -41.746727 20.467390 -32.334212
C -40.761042 19.440262 -30.294274
H -40.436981 18.619559 -29.654854
H -40.309261 20.368144 -29.923843
H -41.846047 19.539252 -30.229195
H -41.110512 17.628509 -36.269041
H -40.067693 16.257385 -36.335408
N -43.966613 17.543176 -29.323986
H -44.040327 17.142896 -28.397249
H -44.620828 18.278197 -29.555326
Cl -39.746312 13.806861 -30.879593
C -38.492670 18.750964 -33.441127
H -38.750547 17.729464 -33.730682
H -39.005728 19.451567 -34.103617
H -37.415128 18.868497 -33.588279
C -38.216612 17.926639 -31.069119
H -38.701707 16.955589 -31.219884
H -37.158390 17.806960 -31.318188
H -38.277762 18.181583 -30.007912
C -38.829777 18.996725 -31.970500
H -38.394713 19.969267 -31.686689

$7_{A4}$
$G_{solv}$= -2438.705006
Ni -41.218911 15.195345 -31.782906
C -42.839481 17.998226 -34.313318
C -41.915098 17.155629 -33.795502
C -43.100989 17.016123 -30.235519
C -41.758793 16.951187 -32.320644
C -42.938512 17.509313 -31.583798
C -43.841117 18.326248 -32.180999
C -41.076654 16.307361 -34.614220
S -40.453485 14.919641 -33.841190
S -42.265030 15.575240 -29.867572
H -42.981331 18.150771 -35.378914
H -44.724595 18.709036 -31.678363
N -40.782729 16.586398 -35.880473
C -44.281175 21.007939 -34.166939
C -44.740085 19.542991 -34.126734
H -44.921895 19.200923 -35.147950
H -45.679478 19.453807 -33.575402
N -43.755841 18.652092 -33.515398
C -44.214670 21.657127 -32.824170
N -43.156107 21.500282 -31.946902
C -45.117044 22.459257 -32.187766
C -43.392519 22.182540 -30.831330
N -44.580964 22.765983 -30.957704
H -46.074744 22.836945 -32.508244
H -42.733972 22.251587 -29.980207
H -43.306021 21.072833 -34.660505
H -44.995349 21.566980 -34.777270
H -45.009503 23.362233 -30.259552
C -40.041559 19.731870 -31.533119
H -40.833981 17.448069 -31.985074
O -40.749625 20.295484 -32.367300
H -42.269065 20.974859 -32.119335
C -40.501884 19.571570 -30.113026
H -40.556578 18.507722 -29.858295
H -39.773552 20.022884 -29.430524
H -41.481491 20.028498 -29.963760
H -41.060122 17.454963 -36.315528
H -40.224966 15.948915 -36.433008
N -43.829760 17.645273 -29.315649
H -43.964631 17.237378 -28.400407
H -44.272469 18.535175 -29.495315
Cl -40.277148 13.263303 -31.030670
C -38.535378 18.977046 -33.412145
H -39.150192 18.114414 -33.691787
H -38.854185 19.835107 -34.008026
H -37.497112 18.744946 -33.665917
C -38.151550 18.063913 -31.079764
H -38.767576 17.172263 -31.241432
H -37.129609 17.817314 -31.381285
H -38.135409 18.281088 -30.008488
C -38.652384 19.249739 -31.912780
H -38.020009 20.118716 -31.665291

$1_B$
$G_{solv}$= -2322.985008
Ni 0.188503 -2.323040 0.391341
C -1.496719 0.318561 -2.263342
C -0.536664 -0.454433 -1.640537
C -1.853878 -0.632254 1.842322
C -0.724180 -0.864420 -0.303842
C -1.764662 -0.242748 0.415396
C -2.693491 0.532276 -0.253222
C 0.646482 -1.045963 -2.309151
S 1.272887 -2.442731 -1.530981
S -1.002367 -2.069369 2.237352
H -1.488550 0.572810 -3.315085
H -3.583383 0.942017 0.204408
N 1.208601 -0.538941 -3.387527
C -2.950261 3.084152 -2.369014
C -3.529707 1.665951 -2.244046
H -3.757263 1.244202 -3.224281

H -4.440993 1.662152 -1.646150
N -2.567696 0.762837 -1.574671
C -2.295275 3.570942 -1.113806
N -0.973388 3.267718 -0.850684
C -2.819575 4.294458 -0.071725
C -0.705312 3.809797 0.319821
N -1.792953 4.435310 0.829449
H -3.796477 4.723180 0.093650
H 0.244858 3.780569 0.832689
H -2.212456 3.101672 -3.177263
H -3.772413 3.742604 -2.662503
H -1.832520 4.940338 1.704439
N -2.502349 0.062811 2.756112
Cl 1.238458 -4.110895 1.203071
C 0.957341 0.810938 -3.905087
H 0.266309 0.778829 -4.752560
H 0.580609 1.466917 -3.121317
H 1.912389 1.212504 -4.246219
C 2.292478 -1.258263 -4.059994
H 3.236268 -1.098022 -3.528169
H 2.074006 -2.325718 -4.098969
H 2.376805 -0.873619 -5.076153
C -2.957650 1.446521 2.589207
H -2.406564 1.945807 1.794166
H -4.032937 1.479536 2.392199
H -2.753823 1.973463 3.523018
C -2.664426 -0.472854 4.108970
H -2.937163 -1.528153 4.070052
H -1.734006 -0.357388 4.674704
H -3.461231 0.083794 4.601710

$6_{B1}$
$G_{solv}$= -2517.226759
Ni 0.098742 -2.693933 0.431628
C -0.775679 0.250477 -2.159371
C 0.075411 -0.561614 -1.492838
C -1.682809 -0.662833 1.851016
C -0.061149 -0.836690 -0.026113
C -1.281152 -0.177861 0.541494
C -2.086074 0.615247 -0.204249
C 1.036197 -1.414735 -2.176540
S 1.246087 -2.956333 -1.440775
S -1.191190 -2.276587 2.182217
H -0.744091 0.397606 -3.232919
H -3.023269 1.019654 0.161602
N 1.721035 -1.054927 -3.257234
C -2.139105 3.237379 -2.288721
C -2.682769 1.800137 -2.270697
H -2.772889 1.441251 -3.299118
H -3.678085 1.780609 -1.820496
N -1.827855 0.883654 -1.528567
C -1.982629 3.830196 -0.926780
N -0.815857 3.734247 -0.190684
C -2.866976 4.513612 -0.142183
C -0.967418 4.345457 0.978682
N -2.210579 4.816222 1.029567
H -3.886161 4.815157 -0.323902
H -0.220026 4.432001 1.751439
H -1.175893 3.260525 -2.808112
H -2.832997 3.856908 -2.863360
H -2.599492 5.336558 1.807207
C 2.149977 1.783777 -0.025115
H 0.839098 -0.506260 0.516157
O 1.519737 2.466188 -0.832482
H 0.066811 3.256676 -0.475817
C 1.802891 1.794459 1.437778
H 2.164160 0.899261 1.947992
H 2.300380 2.661089 1.891489
H 0.724217 1.895244 1.584824
N -2.357963 0.062790 2.740672
Cl 0.553427 -4.821001 1.135088
C 3.299604 0.934845 -0.465144
H 4.149649 1.069304 0.211599
H 2.999941 -0.118296 -0.386225
H 3.588108 1.159400 -1.492234
C 1.822677 0.319209 -3.746239
H 1.201651 0.461802 -4.636493
H 1.528366 1.024456 -2.969975
H 2.865042 0.506950 -4.015982
C 2.552937 -2.022482 -3.967427
H 3.507527 -2.164204 -3.448483
H 2.041716 -2.983311 -4.042793
H 2.742646 -1.642434 -4.971783
C -2.501505 1.514482 2.671520
H -1.780692 1.928459 1.968337
H -3.514631 1.796628 2.366797
H -2.306382 1.925998 3.665479
C -2.876432 -0.545074 3.962692
H -3.271543 -1.540158 3.754289
H -2.088646 -0.624009 4.720371
H -3.681751 0.083754 4.345770

$TS_{6,7-B1}$
$G_{solv}$= -2517.195957

| | | | |
|---|---|---|---|
| Ni | 0.098742 | -2.693933 | 0.431628 |
| C | -0.775679 | 0.250477 | -2.159371 |
| C | 0.075411 | -0.561614 | -1.492838 |
| C | -1.682809 | -0.662833 | 1.851016 |
| C | -0.061149 | -0.836690 | -0.026113 |
| C | -1.281152 | -0.177861 | 0.541494 |
| C | -2.086074 | 0.615247 | -0.204249 |
| C | 1.036197 | -1.414735 | -2.176540 |
| S | 1.246087 | -2.956333 | -1.440775 |
| S | -1.191190 | -2.276587 | 2.182217 |
| H | -0.744091 | 0.397606 | -3.232919 |
| H | -3.023269 | 1.019654 | 0.161602 |
| N | 1.721035 | -1.054927 | -3.257234 |
| C | -2.139105 | 3.237379 | -2.288721 |
| C | -2.682769 | 1.800137 | -2.270697 |
| H | -2.772889 | 1.441251 | -3.299118 |
| H | -3.678085 | 1.780609 | -1.820496 |
| N | -1.827855 | 0.883654 | -1.528567 |
| C | -1.982629 | 3.830196 | -0.926780 |
| N | -0.815857 | 3.734247 | -0.190684 |
| C | -2.866976 | 4.513612 | -0.142183 |
| C | -0.967418 | 4.345457 | 0.978682 |
| N | -2.210579 | 4.816222 | 1.029567 |
| H | -3.886161 | 4.815157 | -0.323902 |
| H | -0.220026 | 4.432001 | 1.751439 |
| H | -1.175893 | 3.260525 | -2.808112 |
| H | -2.832997 | 3.856908 | -2.863360 |
| H | -2.599492 | 5.336558 | 1.807207 |
| C | 2.149977 | 1.783777 | -0.025115 |
| H | 0.839098 | -0.506260 | 0.516157 |
| O | 1.519737 | 2.466188 | -0.832482 |
| H | 0.066811 | 3.256676 | -0.475817 |
| C | 1.802891 | 1.794459 | 1.437778 |
| H | 2.164160 | 0.899261 | 1.947992 |
| H | 2.300380 | 2.661089 | 1.891489 |
| H | 0.724217 | 1.895244 | 1.584824 |
| N | -2.357963 | 0.062790 | 2.740672 |
| Cl | 0.553427 | -4.821001 | 1.135088 |
| C | 3.299604 | 0.934845 | -0.465144 |
| H | 4.149649 | 1.069304 | 0.211599 |
| H | 2.999941 | -0.118296 | -0.386225 |
| H | 3.588108 | 1.159400 | -1.492234 |
| C | 1.822677 | 0.319209 | -3.746239 |
| H | 1.201651 | 0.461802 | -4.636493 |
| H | 1.528366 | 1.024456 | -2.969975 |
| H | 2.865042 | 0.506950 | -4.015982 |
| C | 2.552937 | -2.022482 | -3.967427 |
| H | 3.507527 | -2.164204 | -3.448483 |
| H | 2.041716 | -2.983311 | -4.042793 |
| H | 2.742646 | -1.642434 | -4.971783 |
| C | -2.501505 | 1.514482 | 2.671520 |
| H | -1.780692 | 1.928459 | 1.968337 |
| H | -3.514631 | 1.796628 | 2.366797 |
| H | -2.306382 | 1.925998 | 3.665479 |
| C | -2.876432 | -0.545074 | 3.962692 |
| H | -3.271543 | -1.540158 | 3.754289 |
| H | -2.088646 | -0.624009 | 4.720371 |
| H | -3.681751 | 0.083754 | 4.345770 |

$7_{B2}$

$G_{solv}$= -2536.620504

| | | | |
|---|---|---|---|
| Ni | 0.643752 | -2.564236 | 0.352751 |
| C | -0.929145 | 0.189266 | -2.175021 |
| C | 0.062347 | -0.566925 | -1.645959 |
| C | -1.132419 | -0.663926 | 1.948786 |
| C | 0.193073 | -0.773273 | -0.161749 |
| C | -0.981528 | -0.194948 | 0.580345 |
| C | -1.932022 | 0.539353 | -0.048416 |
| C | 0.924112 | -1.412139 | -2.461633 |
| S | 1.444788 | -2.850955 | -1.674291 |
| S | -0.373170 | -2.176197 | 2.264285 |
| H | -1.095110 | 0.295563 | -3.240842 |
| H | -2.828767 | 0.899516 | 0.440513 |
| N | 1.315019 | -1.140964 | -3.706559 |
| C | -2.300455 | 3.153303 | -2.112145 |
| C | -2.836109 | 1.717295 | -2.014429 |
| H | -3.059257 | 1.345154 | -3.018046 |
| H | -3.764536 | 1.693437 | -1.438098 |
| N | -1.884870 | 0.799450 | -1.395665 |
| C | -2.199873 | 3.881257 | -0.806910 |
| N | -1.136665 | 3.741323 | 0.066678 |
| C | -3.096572 | 4.773878 | -0.277423 |
| C | -1.387489 | 4.542374 | 1.087610 |
| N | -2.564580 | 5.177312 | 0.921774 |
| H | -4.035179 | 5.155302 | -0.649336 |
| H | -0.756372 | 4.681955 | 1.953343 |
| H | -1.320995 | 3.132606 | -2.604893 |
| H | -2.975748 | 3.710721 | -2.769099 |
| H | -2.970327 | 5.851454 | 1.556849 |
| C | 2.006522 | 1.936596 | 0.866677 |
| H | 1.132833 | -0.322121 | 0.197821 |
| H | 0.392329 | 2.850211 | 0.028234 |
| C | 1.384502 | 1.869138 | 2.213514 |
| H | 1.257685 | 0.812858 | 2.481315 |
| H | 2.071325 | 2.301954 | 2.947644 |
| H | 0.423555 | 2.380890 | 2.258302 |

N -1.770381 -0.009190 2.919124
Cl 1.480468 -4.566069 1.077737
C 3.375432 1.388573 0.663489
H 3.779785 0.996260 1.595787
H 3.354528 0.584306 -0.080695
H 4.040345 2.170191 0.278775
N 1.354614 2.452960 -0.119019
C 1.864180 2.558054 -1.478464
H 1.053983 2.923373 -2.109868
H 2.697998 3.264286 -1.517790
H 2.200278 1.581323 -1.835010
C 1.169737 0.151561 -4.371137
H 0.352653 0.125530 -5.099738
H 0.989498 0.943651 -3.648754
H 2.100597 0.369541 -4.900306
C 2.038559 -2.142621 -4.487809
H 3.090582 -2.185824 -4.185227
H 1.589997 -3.128460 -4.354645
H 1.978696 -1.866507 -5.540799
C -2.204246 1.381980 2.841001
H -1.701270 1.897784 2.025828
H -3.287897 1.445016 2.695189
H -1.948659 1.875635 3.782203
C -1.988942 -0.637513 4.219773
H -2.284334 -1.680625 4.096995
H -1.079189 -0.593973 4.829152
H -2.789954 -0.101393 4.729923

$TS_{7,8\text{-}B2}$
$G_{solv}$= -2536.594400
Ni -41.231893 15.463349 -31.920155
C -43.232887 18.160597 -34.253215
C -42.163388 17.467681 -33.760157
C -43.325568 16.890889 -30.232846
C -42.121733 17.061564 -32.365690
C -43.282590 17.460526 -31.585687
C -44.296548 18.184453 -32.150369
C -41.091835 16.912026 -34.594366
S -40.326862 15.522103 -33.934434
S -42.361562 15.481792 -30.025637
H -43.361270 18.391095 -35.302565
H -45.207270 18.449190 -31.629677
N -40.698515 17.425774 -35.750800
C -44.830342 20.757710 -34.447370
C -45.335286 19.365853 -34.050005
H -45.725913 18.842606 -34.926871
H -46.135145 19.442000 -33.312052
N -44.268506 18.537253 -33.462816
C -44.249731 21.552994 -33.319667
N -42.966153 21.346496 -32.846682
C -44.841657 22.565637 -32.608091
C -42.795557 22.224547 -31.876298
N -43.902180 22.978276 -31.696541
H -45.816491 23.023485 -32.680656
H -41.898279 22.343872 -31.286101
H -44.088740 20.663079 -35.249033
H -45.683971 21.295150 -34.871627
H -44.009667 23.727518 -31.026477
C -40.356672 18.841089 -31.560683
H -41.076353 17.827557 -31.863894
H -41.194056 20.231898 -32.821307
C -41.110506 19.379249 -30.360499
H -41.240145 18.604486 -29.600018
H -40.536183 20.201229 -29.919420
H -42.090606 19.763963 -30.653478
N -44.009966 17.403026 -29.221345
Cl -40.061626 13.640722 -31.328723
C -39.038085 18.171675 -31.228794
H -38.321488 18.920626 -30.870692
H -39.188087 17.433052 -30.438015
H -38.608421 17.660213 -32.092928
N -40.345170 19.696654 -32.641556
C -39.277152 19.772921 -33.615770
H -39.522978 20.561495 -34.329090
H -38.325109 20.032020 -33.140520
H -39.131532 18.838212 -34.172204
C -41.095127 18.737766 -36.264929
H -41.904740 18.641028 -36.994857
H -41.396521 19.398050 -35.453084
H -40.228866 19.176994 -36.761768
C -39.714833 16.719422 -36.572046
H -38.706734 16.860422 -36.167873
H -39.940912 15.652585 -36.603401
H -39.759973 17.121464 -37.583909
C -44.614147 18.735467 -29.207826
H -44.137603 19.389150 -29.935669
H -45.689537 18.677490 -29.402311
H -44.460161 19.161231 -28.214624
C -44.092975 16.681106 -27.951035
H -44.284768 15.621377 -28.125624
H -43.158828 16.790982 -27.389660
H -44.915357 17.099516 -27.371336

$7_{B3}$

$G_{solv}$= -2708.865010

| | | | |
|---|---|---|---|
| Ni | -0.083439 | -2.766447 | 0.124144 |
| C | -1.332938 | 0.305660 | -2.284621 |
| C | -0.506226 | -0.654104 | -1.739534 |
| C | -1.710878 | -0.821104 | 1.767761 |
| C | -0.742311 | -1.126158 | -0.435672 |
| C | -1.634363 | -0.378286 | 0.356923 |
| C | -2.442646 | 0.577202 | -0.231183 |
| C | 0.558253 | -1.380010 | -2.467003 |
| S | 0.866224 | -2.959480 | -1.873711 |
| S | -1.146903 | -2.420006 | 2.038257 |
| H | -1.299308 | 0.620591 | -3.320197 |
| H | -3.249290 | 1.087633 | 0.279296 |
| N | 1.243223 | -0.851983 | -3.459346 |
| C | -2.689239 | 3.241950 | -2.250771 |
| C | -3.244918 | 1.816456 | -2.190628 |
| H | -3.424751 | 1.440736 | -3.199016 |
| H | -4.185558 | 1.779722 | -1.639647 |
| N | -2.315673 | 0.860692 | -1.543365 |
| C | -2.641806 | 3.962592 | -0.939837 |
| N | -1.662736 | 3.724365 | 0.004806 |
| C | -3.491852 | 4.933306 | -0.475105 |
| C | -1.913890 | 4.542419 | 1.009579 |
| N | -3.013324 | 5.284977 | 0.762360 |
| H | -4.360739 | 5.400707 | -0.912651 |
| H | -1.335886 | 4.623605 | 1.918518 |
| H | -1.692607 | 3.220701 | -2.705955 |
| H | -3.338900 | 3.796599 | -2.934171 |
| H | -3.398536 | 5.995829 | 1.369592 |
| C | 4.010885 | -0.113839 | -1.231972 |
| C | 4.165125 | -1.257998 | -0.448347 |
| C | 3.334585 | -1.454881 | 0.653232 |
| C | 2.352792 | -0.515018 | 0.963633 |
| C | 2.189741 | 0.631294 | 0.182861 |
| C | 3.031750 | 0.826165 | -0.915123 |
| H | 4.657422 | 0.048670 | -2.090168 |
| H | 4.922364 | -1.995053 | -0.698792 |
| H | 3.436058 | -2.349312 | 1.260439 |
| H | 1.699129 | -0.683670 | 1.816389 |
| H | 2.914366 | 1.717850 | -1.523148 |
| C | 1.148415 | 1.666248 | 0.549090 |
| H | 0.377033 | 1.162393 | 1.152379 |
| O | 0.566769 | 2.170118 | -0.641957 |
| H | -0.180391 | 2.757122 | -0.389215 |
| C | 1.748522 | 2.793300 | 1.390537 |
| H | 2.182394 | 2.396989 | 2.314798 |
| H | 2.534787 | 3.309873 | 0.830133 |
| H | 0.975132 | 3.522472 | 1.657258 |
| N | -2.122869 | -0.051504 | 2.755338 |
| Cl | 0.649814 | -4.767623 | 0.781039 |
| C | 1.249197 | 0.576803 | -3.787658 |
| H | 0.632626 | 0.770621 | -4.670064 |
| H | 0.918193 | 1.170925 | -2.935583 |
| H | 2.279616 | 0.855360 | -4.014260 |
| C | 2.211403 | -1.663336 | -4.196401 |
| H | 3.118774 | -1.796647 | -3.597359 |
| H | 1.787276 | -2.639912 | -4.433825 |
| H | 2.456772 | -1.144566 | -5.122820 |
| C | -2.310459 | 1.397822 | 2.656416 |
| H | -1.776805 | 1.804882 | 1.800225 |
| H | -3.373981 | 1.645928 | 2.591315 |
| H | -1.897844 | 1.847186 | 3.561425 |
| C | -2.264398 | -0.599668 | 4.105361 |
| H | -2.752065 | -1.574351 | 4.070726 |
| H | -1.280823 | -0.702009 | 4.575715 |
| H | -2.877748 | 0.085781 | 4.689367 |

$TS_{2,3\text{-}B}$

$G_{solv}$= -2406.130651

| | | | |
|---|---|---|---|
| Ni | -40.187958 | 16.295046 | -32.359634 |
| C | -43.401321 | 18.108004 | -34.103027 |
| C | -42.116188 | 17.671837 | -33.979517 |
| C | -41.666782 | 17.919466 | -30.257782 |
| C | -41.469493 | 17.627981 | -32.683346 |
| C | -42.275285 | 18.105956 | -31.580052 |
| C | -43.561601 | 18.528920 | -31.788189 |
| S | -40.165454 | 15.922518 | -34.542033 |
| S | -40.440909 | 16.717911 | -30.202361 |
| H | -43.957064 | 18.081579 | -35.032265 |
| H | -44.233179 | 18.813267 | -30.988952 |
| C | -45.503050 | 20.456024 | -33.779618 |
| C | -45.485809 | 19.026642 | -33.229838 |
| H | -45.977874 | 18.349913 | -33.932105 |
| H | -46.008696 | 18.963024 | -32.273868 |
| N | -44.116667 | 18.529898 | -33.022980 |
| C | -44.994413 | 21.502086 | -32.837361 |
| N | -43.661502 | 21.618855 | -32.485060 |
| C | -45.716264 | 22.489147 | -32.216225 |
| C | -43.592009 | 22.661849 | -31.676879 |
| N | -44.808443 | 23.213010 | -31.485091 |
| H | -46.766172 | 22.737955 | -32.243202 |
| H | -42.693033 | 23.045623 | -31.215976 |
| H | -44.930473 | 20.484171 | -34.714360 |
| H | -46.540531 | 20.688844 | -34.037587 |
| H | -45.010114 | 24.027567 | -30.920884 |
| B | -39.959689 | 19.793014 | -33.173390 |

H -38.798571 19.865564 -32.853095
H -40.347393 18.610476 -32.814785
H -40.219792 19.871343 -34.351589
N -40.828462 20.805247 -32.331263
H -40.463263 21.750706 -32.454936
H -41.834243 20.848560 -32.576943
H -40.768067 20.603413 -31.331617
C -41.330582 17.070598 -35.064755
N -41.467327 17.395257 -36.339200
N -41.990893 18.624483 -29.183861
Cl -38.602387 14.759102 -31.975467
C -40.754477 16.647809 -37.374491
H -40.767070 15.580552 -37.151297
H -41.258434 16.818631 -38.326056
H -39.716948 16.991820 -37.443788
C -42.152764 18.597907 -36.817746
H -43.142047 18.352395 -37.215118
H -42.234447 19.337750 -36.022649
H -41.548030 19.025349 -37.619866
C -42.776711 19.859029 -29.198155
H -42.810783 20.290797 -30.196706
H -43.794783 19.675258 -28.841132
H -42.291724 20.571235 -28.527095
C -41.453827 18.261852 -27.872115
H -42.072402 18.731597 -27.107279
H -41.481000 17.179900 -27.735363
H -40.421133 18.612267 -27.770823

$5_B$
$G_{solv}$= -2324.167267
Ni -41.164407 15.872364 -31.841578
C -43.009376 18.412274 -34.349659
C -41.928878 17.930804 -33.693031
C -43.580242 17.046572 -30.402729
C -41.969598 17.575459 -32.232976
C -43.360861 17.710565 -31.679845
C -44.386994 18.195554 -32.416715
C -40.741611 17.468798 -34.403383
S -40.008557 16.078291 -33.709954
S -42.521470 15.725573 -30.106925
H -43.023903 18.598468 -35.417681
H -45.411692 18.218485 -32.063495
N -40.240240 18.051342 -35.488839
C -45.045399 20.854603 -34.381940
C -45.283225 19.332736 -34.406176
H -45.344656 18.992954 -35.443164
H -46.229948 19.093265 -33.917718
N -44.223918 18.604515 -33.722893
C -44.766659 21.370465 -33.009706
N -43.495631 21.368837 -32.460624
C -45.584637 21.862739 -32.035884
C -43.523750 21.846113 -31.219957
N -44.788749 22.145047 -30.947202
H -46.647424 22.043823 -32.032326
H -42.680042 21.961678 -30.559038
H -44.203782 21.108004 -35.035052
H -45.929977 21.356397 -34.781891
H -45.108821 22.545359 -30.072453
H -42.655296 21.044927 -32.927922
H -41.266928 18.210802 -31.666336
Cl -39.996625 14.011261 -31.209586
N -44.501809 17.410474 -29.513408
C -40.595937 19.392639 -35.945057
H -41.252999 19.349075 -36.819734
H -41.083894 19.950415 -35.146803
H -39.675864 19.912076 -36.223907
C -39.159151 17.418637 -36.240694
H -38.194388 17.589108 -35.750215
H -39.331683 16.344623 -36.324291
H -39.136315 17.851954 -37.241205
C -45.209323 18.687807 -29.533133
H -44.694996 19.397557 -30.178426
H -46.241111 18.561129 -29.876487
H -45.226337 19.083521 -28.514279
C -44.770503 16.585354 -28.338500
H -44.760892 15.527526 -28.604654
H -44.021695 16.764933 -27.559076
H -45.757931 16.844736 -27.954487

$TS_{6,7\text{-}B4}$
$G_{solv}$= -2595.753388
Ni -41.120788 15.453145 -31.794429
C -43.079344 18.006144 -34.275517
C -42.013075 17.351136 -33.749670
C -43.127043 17.064475 -30.161998
C -41.882931 17.122926 -32.302912
C -43.078061 17.545956 -31.542479
C -44.110732 18.189838 -32.156009
C -41.018749 16.663195 -34.573250
S -40.395895 15.222113 -33.874486
S -42.128959 15.698325 -29.850420
H -43.229047 18.144281 -35.340024
H -45.030744 18.456414 -31.652677
N -40.588623 17.118615 -35.738967

C -44.600882 20.764071 -34.264212
C -45.118087 19.330218 -34.090044
H -45.393353 18.914090 -35.062379
H -46.001803 19.310942 -33.449238
N -44.102499 18.459627 -33.492216
C -44.254628 21.460725 -32.984895
N -43.072046 21.246911 -32.296483
C -44.995714 22.393890 -32.308266
C -43.102759 22.045729 -31.243160
N -44.249988 22.747936 -31.211436
H -45.957760 22.835024 -32.519179
H -42.328260 22.132810 -30.495300
H -43.727094 20.753044 -34.925819
H -45.381981 21.333308 -34.776935
H -44.504483 23.434183 -30.513232
C -40.353232 19.187007 -31.798342
H -40.938126 17.819727 -31.943766
O -40.866425 19.883164 -32.786061
H -41.756743 20.352533 -32.575933
C -40.848752 19.474587 -30.408271
H -40.631413 18.655447 -29.722261
H -40.327703 20.371165 -30.050638
H -41.919074 19.677985 -30.402692
N -43.848033 17.611368 -29.189704
Cl -40.091027 13.559988 -31.131950
C -38.470783 18.530854 -33.405255
H -38.752985 17.507527 -33.663460
H -38.913571 19.222004 -34.125926
H -37.382573 18.599996 -33.491716
C -38.368995 17.792162 -30.994494
H -38.891471 16.840145 -31.140849
H -37.305371 17.615780 -31.177624
H -38.478544 18.090443 -29.948620
C -38.878121 18.854652 -31.968460
H -38.408320 19.814926 -31.697583
C -44.491371 18.923651 -29.253426
H -44.045244 19.543517 -30.029726
H -45.566195 18.821885 -29.433947
H -44.342775 19.418352 -28.291362
C -43.943738 16.952820 -27.886477
H -44.097953 15.879987 -28.011491
H -43.030901 17.118872 -27.304275
H -44.795800 17.371837 -27.351042
C -40.821880 18.478689 -36.227589
H -41.600972 18.485988 -36.995972
H -41.095183 19.137066 -35.404064
H -39.890383 18.839389 -36.668806
C -39.675925 16.322547 -36.556911
H -38.651573 16.414507 -36.178469
H -39.970009 15.272678 -36.547822
H -39.718862 16.695298 -37.580466

$6_{C1}$
$G_{solv}$= -2111.038668
Ni 0.216364 -2.657190 0.509455
C -0.716279 0.288569 -2.196642
C 0.094946 -0.583006 -1.535494
C -1.697129 -0.574719 1.818604
C -0.040454 -0.822172 -0.064967
C -1.250450 -0.155033 0.478349
C -2.004421 0.692930 -0.245167
C 1.014955 -1.462620 -2.211417
S 1.238306 -2.991372 -1.421017
S -1.034290 -2.158710 2.261552
H -0.662091 0.442110 -3.268167
H -2.902109 1.152785 0.154532
N 1.690067 -1.167179 -3.316896
C -2.053752 3.354934 -2.254551
C -2.559918 1.905414 -2.321745
H -2.589362 1.584984 -3.365384
H -3.576330 1.843806 -1.924928
N -1.719716 0.974028 -1.573701
C -1.997155 3.889721 -0.861948
N -0.908368 3.696330 -0.031997
C -2.926707 4.555275 -0.117138
C -1.152221 4.222669 1.162253
N -2.374354 4.746236 1.129415
H -3.916610 4.902026 -0.366608
H -0.481420 4.220971 2.006554
H -1.063090 3.422093 -2.715397
H -2.731477 3.978280 -2.843937
H -2.822240 5.212761 1.909595
C 2.095062 1.786107 -0.026301
H 0.864604 -0.496541 0.473625
O 1.408494 2.457142 -0.795915
H -0.018507 3.221817 -0.296677
C 1.812999 1.770652 1.449810
H 2.215082 0.877705 1.932956
H 2.310898 2.642513 1.893052
H 0.739451 1.847193 1.643147
C 3.245070 0.973635 -0.531632
H 4.120842 1.114784 0.109800
H 2.970853 -0.087287 -0.462907
H 3.483299 1.224063 -1.565676
C 1.833755 0.188124 -3.848273
H 1.183990 0.337432 -4.716576

| | | | |
|---|---|---|---|
| H | 1.602553 | 0.926405 | -3.081126 |
| H | 2.871521 | 0.319701 | -4.163874 |
| C | 2.464244 | -2.186593 | -4.022310 |
| H | 3.442293 | -2.324944 | -3.548650 |
| H | 1.929361 | -3.137148 | -4.029516 |
| H | 2.607901 | -1.856815 | -5.051751 |
| O | -2.444608 | 0.065100 | 2.547222 |
| C | 0.648303 | -4.284036 | 1.187802 |
| O | 0.903433 | -5.301735 | 1.635203 |

$TS_{6,7\text{-}C1}$

$G_{solv}$= -2111.006870

| | | | |
|---|---|---|---|
| Ni | -41.455603 | 15.260564 | -31.620231 |
| C | -43.047010 | 17.915979 | -34.366761 |
| C | -42.120471 | 17.114803 | -33.760071 |
| C | -43.602680 | 17.032241 | -30.277612 |
| C | -42.144024 | 16.913935 | -32.308072 |
| C | -43.343028 | 17.445540 | -31.671981 |
| C | -44.206098 | 18.252797 | -32.335388 |
| C | -41.111089 | 16.332743 | -34.460207 |
| S | -40.614089 | 14.893000 | -33.630007 |
| S | -42.556781 | 15.692029 | -29.761861 |
| H | -43.085375 | 18.076981 | -35.437322 |
| H | -45.085230 | 18.676238 | -31.861713 |
| N | -40.549607 | 16.670030 | -35.607116 |
| C | -44.181560 | 20.887723 | -34.388935 |
| C | -44.880269 | 19.522169 | -34.337403 |
| H | -45.087801 | 19.169947 | -35.350438 |
| H | -45.826187 | 19.587648 | -33.796358 |
| N | -44.046662 | 18.517626 | -33.669164 |
| C | -43.926605 | 21.492861 | -33.043843 |
| N | -42.858122 | 21.129754 | -32.241877 |
| C | -44.670467 | 22.435439 | -32.384740 |
| C | -42.959146 | 21.843741 | -31.134255 |
| N | -44.040857 | 22.641041 | -31.182196 |
| H | -45.566000 | 22.967946 | -32.665575 |
| H | -42.283014 | 21.803109 | -30.293282 |
| H | -43.236880 | 20.785709 | -34.934809 |
| H | -44.819143 | 21.561168 | -34.969266 |
| H | -44.332945 | 23.283152 | -30.457470 |
| C | -40.420201 | 18.772815 | -31.760878 |
| H | -41.188104 | 17.561014 | -31.876595 |
| O | -40.827796 | 19.560698 | -32.729404 |
| H | -41.651545 | 20.149159 | -32.500782 |
| C | -40.813466 | 19.107378 | -30.350929 |
| H | -40.532172 | 18.303669 | -29.669197 |
| H | -40.279659 | 20.020093 | -30.058945 |
| H | -41.886793 | 19.296807 | -30.265949 |
| C | -39.077705 | 18.146818 | -32.009785 |
| H | -38.310774 | 18.911774 | -31.835598 |
| H | -38.899914 | 17.312687 | -31.328453 |
| H | -38.992029 | 17.807435 | -33.045156 |
| C | -40.636572 | 18.006880 | -36.199961 |
| H | -41.376883 | 18.027932 | -37.004838 |
| H | -40.878237 | 18.745791 | -35.437384 |
| H | -39.657565 | 18.248086 | -36.617743 |
| C | -39.643923 | 15.752623 | -36.300788 |
| H | -38.639069 | 15.816655 | -35.870027 |
| H | -40.007022 | 14.727267 | -36.229745 |
| H | -39.606151 | 16.040226 | -37.351651 |
| O | -44.441676 | 17.533181 | -29.547472 |
| C | -40.721217 | 13.757988 | -30.896312 |
| O | -40.270326 | 12.823850 | -30.429947 |

$7_{C2}$

$G_{solv}$= -2130.435685

| | | | |
|---|---|---|---|
| Ni | 0.743333 | -2.551981 | 0.428483 |
| C | -0.801001 | 0.251497 | -2.185031 |
| C | 0.130348 | -0.602832 | -1.667489 |
| C | -1.018351 | -0.499433 | 1.959866 |
| C | 0.295158 | -0.766263 | -0.184724 |
| C | -0.825097 | -0.119662 | 0.549648 |
| C | -1.711598 | 0.699774 | -0.045332 |
| C | 0.875060 | -1.535451 | -2.475271 |
| S | 1.315176 | -2.996911 | -1.647957 |
| S | -0.167248 | -2.002862 | 2.361417 |
| H | -0.962741 | 0.364051 | -3.250535 |
| H | -2.534500 | 1.152063 | 0.496573 |
| N | 1.251395 | -1.353065 | -3.738593 |
| C | -2.227213 | 3.322438 | -2.021841 |
| C | -2.654676 | 1.847841 | -2.018554 |
| H | -2.815129 | 1.513387 | -3.046450 |
| H | -3.599226 | 1.730639 | -1.480589 |
| N | -1.668779 | 0.955958 | -1.406621 |
| C | -2.185950 | 3.953443 | -0.665204 |
| N | -1.128309 | 3.782238 | 0.207208 |
| C | -3.143742 | 4.724497 | -0.058934 |
| C | -1.445099 | 4.434697 | 1.310756 |
| N | -2.654718 | 5.016604 | 1.190426 |
| H | -4.102541 | 5.082294 | -0.401543 |
| H | -0.838476 | 4.503688 | 2.201599 |
| H | -1.250203 | 3.414105 | -2.510734 |
| H | -2.948099 | 3.865472 | -2.641203 |
| H | -3.116195 | 5.576321 | 1.894749 |

| | | | |
|---|---|---|---|
| C | 2.024975 | 2.039735 | 0.811975 |
| H | 1.271779 | -0.366401 | 0.141441 |
| H | 0.370989 | 2.916206 | 0.025962 |
| C | 1.423030 | 1.939500 | 2.166016 |
| H | 1.498290 | 0.901650 | 2.509270 |
| H | 2.016881 | 2.542813 | 2.861745 |
| H | 0.380950 | 2.257105 | 2.191722 |
| C | 3.415681 | 1.555134 | 0.588477 |
| H | 3.868521 | 1.230482 | 1.524933 |
| H | 3.408639 | 0.711979 | -0.112459 |
| H | 4.027668 | 2.344690 | 0.139399 |
| N | 1.336771 | 2.533078 | -0.160038 |
| C | 1.816426 | 2.674739 | -1.526748 |
| H | 0.971431 | 2.970979 | -2.148186 |
| H | 2.590106 | 3.445885 | -1.580152 |
| H | 2.224136 | 1.728109 | -1.888119 |
| C | 1.404784 | -4.104758 | 1.095354 |
| O | 1.827263 | -5.068165 | 1.536381 |
| O | -1.687020 | 0.122527 | 2.775021 |
| C | 1.834567 | -2.444742 | -4.516848 |
| H | 1.715348 | -2.212030 | -5.575802 |
| H | 2.900256 | -2.556603 | -4.289597 |
| H | 1.321118 | -3.383556 | -4.304585 |
| C | 1.227702 | -0.063839 | -4.423610 |
| H | 0.363676 | 0.010222 | -5.092089 |
| H | 1.207452 | 0.751113 | -3.701831 |
| H | 2.138471 | 0.020716 | -5.020694 |

$TS_{7,8\text{-}C2}$

$G_{solv}$= -2130.405100

| | | | |
|---|---|---|---|
| Ni | -41.005997 | 15.550675 | -31.843190 |
| C | -43.261755 | 18.174775 | -34.134606 |
| C | -42.172809 | 17.451356 | -33.712041 |
| C | -43.190323 | 16.899163 | -30.118706 |
| C | -42.048590 | 17.068209 | -32.321155 |
| C | -43.168994 | 17.435776 | -31.500541 |
| C | -44.182159 | 18.215694 | -31.969187 |
| C | -41.148130 | 16.894525 | -34.591115 |
| S | -40.306785 | 15.529184 | -33.939239 |
| S | -41.973280 | 15.634018 | -29.864603 |
| H | -43.452312 | 18.411391 | -35.172488 |
| H | -45.017389 | 18.523305 | -31.350009 |
| N | -40.814372 | 17.370249 | -35.777087 |
| C | -44.855514 | 20.802980 | -34.240253 |
| C | -45.327324 | 19.412061 | -33.803598 |
| H | -45.774821 | 18.881305 | -34.647925 |
| H | -46.074137 | 19.488546 | -33.012451 |
| N | -44.225201 | 18.585003 | -33.280990 |
| C | -44.223316 | 21.608663 | -33.148651 |
| N | -42.889606 | 21.479279 | -32.806741 |
| C | -44.813161 | 22.547906 | -32.341416 |
| C | -42.687178 | 22.332023 | -31.819616 |
| N | -43.821310 | 22.992934 | -31.503667 |
| H | -45.821096 | 22.931725 | -32.299064 |
| H | -41.751021 | 22.489071 | -31.303682 |
| H | -44.156516 | 20.709654 | -35.079336 |
| H | -45.736700 | 21.328348 | -34.620977 |
| H | -43.918030 | 23.699542 | -30.787299 |
| C | -40.289301 | 18.921536 | -31.661807 |
| H | -40.988270 | 17.877613 | -31.884568 |
| H | -41.208756 | 20.300259 | -32.881980 |
| C | -40.997799 | 19.470580 | -30.439444 |
| H | -41.042867 | 18.716980 | -29.648954 |
| H | -40.438811 | 20.333101 | -30.060443 |
| H | -42.014448 | 19.795766 | -30.675678 |
| C | -38.927573 | 18.316341 | -31.378583 |
| H | -38.226155 | 19.105076 | -31.081324 |
| H | -39.008789 | 17.600493 | -30.556999 |
| H | -38.518653 | 17.796378 | -32.248107 |
| N | -40.374295 | 19.721429 | -32.779633 |
| C | -39.344765 | 19.838679 | -33.789950 |
| H | -39.665622 | 20.586608 | -34.517075 |
| H | -38.393091 | 20.170439 | -33.359818 |
| H | -39.162310 | 18.897716 | -34.324821 |
| C | -39.932151 | 14.174899 | -31.317059 |
| O | -39.264427 | 13.323429 | -30.968610 |
| O | -43.957246 | 17.267945 | -29.246925 |
| C | -39.864059 | 16.650066 | -36.628330 |
| H | -38.839763 | 16.811544 | -36.277567 |
| H | -40.082679 | 15.581203 | -36.624258 |
| H | -39.962271 | 17.027430 | -37.645594 |
| C | -41.274713 | 18.650695 | -36.317885 |
| H | -42.137931 | 18.502819 | -36.973488 |
| H | -41.522990 | 19.341321 | -35.513112 |
| H | -40.458147 | 19.077819 | -36.900553 |

$6_{C3}$

$G_{solv}$= -2302.675070

| | | | |
|---|---|---|---|
| Ni | -0.414822 | -2.662306 | 0.437064 |
| C | -1.224280 | 0.299938 | -2.317651 |
| C | -0.460408 | -0.598930 | -1.636636 |
| C | -2.374277 | -0.566020 | 1.654621 |
| C | -0.661245 | -0.839606 | -0.173634 |
| C | -1.878101 | -0.155918 | 0.329482 |
| C | -2.583515 | 0.716330 | -0.414327 |
| C | 0.468658 | -1.500262 | -2.275471 |

| | | | |
|---|---|---|---|
| S | 0.646186 | -3.020970 | -1.461188 |
| S | -1.727090 | -2.144598 | 2.137729 |
| H | -1.130075 | 0.461513 | -3.385269 |
| H | -3.486548 | 1.193551 | -0.047857 |
| N | 1.179285 | -1.241393 | -3.368274 |
| C | -2.422234 | 3.417598 | -2.339945 |
| C | -2.987483 | 1.995668 | -2.485712 |
| H | -2.966957 | 1.710475 | -3.539479 |
| H | -4.028031 | 1.967439 | -2.153106 |
| N | -2.235427 | 1.003107 | -1.725369 |
| C | -2.556056 | 3.971940 | -0.959386 |
| N | -1.628333 | 3.760997 | 0.045763 |
| C | -3.566705 | 4.693145 | -0.393037 |
| C | -2.046818 | 4.329662 | 1.170867 |
| N | -3.222458 | 4.897893 | 0.923783 |
| H | -4.484231 | 5.072329 | -0.813390 |
| H | -1.529126 | 4.321251 | 2.116603 |
| H | -1.370957 | 3.428377 | -2.644527 |
| H | -2.969225 | 4.069583 | -3.026382 |
| H | -3.773324 | 5.401564 | 1.609089 |
| C | 3.904305 | -0.044154 | -1.355365 |
| C | 4.347140 | -1.036396 | -0.479571 |
| C | 3.821562 | -1.117215 | 0.809850 |
| C | 2.848473 | -0.213227 | 1.223400 |
| C | 2.388683 | 0.776642 | 0.343739 |
| C | 2.929296 | 0.857575 | -0.945438 |
| H | 4.319554 | 0.023730 | -2.356226 |
| H | 5.102404 | -1.746719 | -0.802046 |
| H | 4.164170 | -1.889732 | 1.490857 |
| H | 2.437023 | -0.299558 | 2.223776 |
| H | 2.575224 | 1.631561 | -1.617618 |
| C | 1.286872 | 1.703430 | 0.722738 |
| H | 0.231027 | -0.529371 | 0.388756 |
| O | 0.815573 | 2.469021 | -0.121066 |
| H | -0.722502 | 3.257965 | -0.035027 |
| C | 0.749858 | 1.675386 | 2.125341 |
| H | 0.294370 | 0.701647 | 2.338589 |
| H | 1.558733 | 1.822530 | 2.847853 |
| H | -0.001811 | 2.453083 | 2.259902 |
| C | 1.323721 | 0.086827 | -3.958333 |
| H | 0.684784 | 0.192025 | -4.841431 |
| H | 1.076761 | 0.857814 | -3.230522 |
| H | 2.364441 | 0.213107 | -4.264949 |
| C | 1.974735 | -2.285693 | -4.010712 |
| H | 2.916269 | -2.440069 | -3.472482 |
| H | 1.421189 | -3.225478 | -4.041986 |
| H | 2.190266 | -1.972758 | -5.032680 |
| O | -3.146995 | 0.080723 | 2.350494 |
| C | 0.027429 | -4.260182 | 1.171822 |
| O | 0.292201 | -5.253349 | 1.666984 |

$TS_{6,7\text{-}C3}$

$G_{solv}$= -2302.643656

| | | | |
|---|---|---|---|
| Ni | -41.276609 | 15.269546 | -31.875060 |
| C | -43.096519 | 17.981930 | -34.428129 |
| C | -42.125278 | 17.174097 | -33.904232 |
| C | -43.391401 | 16.935980 | -30.347072 |
| C | -42.075261 | 16.909395 | -32.468327 |
| C | -43.237625 | 17.394170 | -31.744543 |
| C | -44.150017 | 18.212591 | -32.325883 |
| C | -41.143041 | 16.437372 | -34.690950 |
| S | -40.594017 | 14.967589 | -33.957449 |
| S | -42.279222 | 15.612184 | -29.941554 |
| H | -43.190096 | 18.190865 | -35.487453 |
| H | -45.004843 | 18.607098 | -31.787297 |
| N | -40.664173 | 16.825663 | -35.858700 |
| C | -44.287206 | 20.934398 | -34.239069 |
| C | -44.953634 | 19.551991 | -34.228531 |
| H | -45.203559 | 19.248218 | -35.247669 |
| H | -45.872863 | 19.566683 | -33.639821 |
| N | -44.067873 | 18.532729 | -33.653504 |
| C | -43.966976 | 21.467914 | -32.877404 |
| N | -42.843910 | 21.091790 | -32.159718 |
| C | -44.697765 | 22.347862 | -32.123753 |
| C | -42.902069 | 21.737905 | -31.008009 |
| N | -44.007125 | 22.502565 | -30.947875 |
| H | -45.621969 | 22.868093 | -32.323450 |
| H | -42.178597 | 21.670816 | -30.209215 |
| H | -43.375619 | 20.888684 | -34.845191 |
| H | -44.973162 | 21.623451 | -34.740601 |
| H | -44.275166 | 23.095080 | -30.173457 |
| C | -37.355164 | 17.668771 | -34.027160 |
| C | -36.750517 | 16.733324 | -33.189587 |
| C | -37.286849 | 16.484008 | -31.924760 |
| C | -38.426217 | 17.158106 | -31.504140 |
| C | -39.044839 | 18.093290 | -32.345799 |
| C | -38.499437 | 18.343764 | -33.610227 |
| H | -36.935043 | 17.875121 | -35.006842 |
| H | -35.862399 | 16.201600 | -33.517098 |
| H | -36.818351 | 15.758044 | -31.267573 |
| H | -38.840817 | 16.934958 | -30.526380 |
| H | -38.968609 | 19.073630 | -34.259915 |
| C | -40.320437 | 18.748093 | -31.949069 |
| H | -41.098274 | 17.558097 | -32.057788 |

O -40.806240 19.578299 -32.853410
H -41.612272 20.138701 -32.539093
C -40.623115 19.045239 -30.506749
H -40.332211 18.229747 -29.845964
H -40.069522 19.946482 -30.219014
H -41.690701 19.236352 -30.372939
C -40.800080 18.183809 -36.388662
H -41.566818 18.220929 -37.167994
H -41.033908 18.886157 -35.589807
H -39.840549 18.463612 -36.828587
C -39.786082 15.951663 -36.636536
H -38.766182 15.992745 -36.238968
H -40.146060 14.922990 -36.608895
H -39.787561 16.298450 -37.669811
C -40.343511 13.843693 -31.226923
O -39.725571 12.989650 -30.799930
O -44.189971 17.396082 -29.548145

$5_C$

$G_{solv}$= -1917.981679

Ni -41.017355 15.905348 -31.834389
C -42.868482 18.496189 -34.434590
C -41.813222 17.894721 -33.816818
C -43.456022 17.232971 -30.444372
C -41.827199 17.598272 -32.344119
C -43.185552 17.813591 -31.772981
C -44.182366 18.400889 -32.458431
C -40.705210 17.323378 -34.545671
S -40.009444 15.924569 -33.793378
S -42.301738 15.952442 -30.037846
H -42.896380 18.684896 -35.501999
H -45.176471 18.529753 -32.043624
N -40.196581 17.797708 -35.677872
C -44.940240 21.086137 -34.250311
C -45.080306 19.565197 -34.438135
H -45.056013 19.332243 -35.505072
H -46.038157 19.223066 -34.039121
N -44.017925 18.821215 -33.771579
C -44.924044 21.489110 -32.813296
N -43.764832 21.515355 -32.057524
C -45.931340 21.802341 -31.949177
C -44.040397 21.826901 -30.794926
N -45.353597 22.004236 -30.715274
H -46.993747 21.893598 -32.108081
H -43.331230 21.917247 -29.988027
H -44.023860 21.434457 -34.737768
H -45.782630 21.574442 -34.746836
H -45.848985 22.249133 -29.865549
H -42.832998 21.320311 -32.406242
H -41.073961 18.212265 -31.819738
C -40.475881 19.133422 -36.201532
H -41.214557 19.092941 -37.008496
H -40.833372 19.787025 -35.406683
H -39.545356 19.544156 -36.598969
C -39.193074 17.044715 -36.429098
H -38.193591 17.214261 -36.014209
H -39.416894 15.977680 -36.404154
H -39.215078 17.383853 -37.465392
C -40.124241 14.445458 -31.221713
O -39.586777 13.530896 -30.803585
O -44.366422 17.571985 -29.699241

$TS_{6,7\text{-}C4}$

$G_{solv}$= -2189.565841

Ni -41.026639 15.514846 -31.759638
C -43.042634 18.049549 -34.330555
C -42.007115 17.329098 -33.805489
C -43.221977 17.060440 -30.236770
C -41.892164 17.116322 -32.360337
C -43.088990 17.524652 -31.633810
C -44.071031 18.255293 -32.218520
C -41.035097 16.604912 -34.609255
S -40.398857 15.180917 -33.855940
S -42.023093 15.821151 -29.816729
H -43.175605 18.204964 -35.394730
H -44.950284 18.583344 -31.674610
N -40.601337 16.987435 -35.795840
C -44.477837 20.902956 -34.231592
C -45.017158 19.469098 -34.148292
H -45.257585 19.101426 -35.148766
H -45.924139 19.428400 -33.542264
N -44.035855 18.554910 -33.553473
C -44.223890 21.535687 -32.899649
N -43.097653 21.268243 -32.141727
C -45.020066 22.413988 -32.213310
C -43.215705 21.976647 -31.031977
N -44.363391 22.677903 -31.036908
H -45.968596 22.865949 -32.459177
H -42.506838 21.996693 -30.217284
H -43.557714 20.905898 -34.826844
H -45.216673 21.499484 -34.774868
H -44.681036 23.295625 -30.301963
C -40.392884 19.174860 -31.787010
H -40.947117 17.845373 -31.989557
O -40.923936 19.915526 -32.736999
H -41.800174 20.391178 -32.474548

| | | | |
|---|---|---|---|
| C | -40.883504 | 19.394312 | -30.383430 |
| H | -40.620606 | 18.563431 | -29.728901 |
| H | -40.406305 | 20.303883 | -29.999451 |
| H | -41.964578 | 19.538110 | -30.360160 |
| C | -38.537134 | 18.550551 | -33.433996 |
| H | -38.793891 | 17.514799 | -33.669929 |
| H | -39.026653 | 19.216139 | -34.148657 |
| H | -37.455463 | 18.654223 | -33.559602 |
| C | -38.342995 | 17.847831 | -31.019030 |
| H | -38.841650 | 16.877918 | -31.136427 |
| H | -37.280473 | 17.699370 | -31.232295 |
| H | -38.430992 | 18.156014 | -29.974081 |
| C | -38.911491 | 18.882548 | -31.989371 |
| H | -38.461115 | 19.857896 | -31.742992 |
| C | -40.806617 | 18.327099 | -36.350649 |
| H | -41.599620 | 18.317535 | -37.104338 |
| H | -41.042162 | 19.037317 | -35.559410 |
| H | -39.873233 | 18.632972 | -36.827420 |
| C | -39.731291 | 16.120981 | -36.592035 |
| H | -38.699721 | 16.184719 | -36.229768 |
| H | -40.071848 | 15.086256 | -36.541610 |
| H | -39.771924 | 16.455811 | -37.628379 |
| C | -40.078971 | 14.101046 | -31.108436 |
| O | -39.470392 | 13.243267 | -30.675748 |
| O | -44.058304 | 17.464161 | -29.445383 |

## References


1. A. D. Becke, *J. Chem. Phys.*, 1993, 98, 5648–5652.

2. C. Lee, W. Yang and R. G. Parr, *Phys. Rev. B*, 1988, 37, 785–789.

3. L. Goerigk and S. Grimme, *Phys. Chem. Chem. Phys.*, 2011, 13, 6670−6688.

4. Y. Zhao and D. G. Truhlar, *Theor. Chem. Acc.*, 2008, 120, 215–241.

5. J. Tao, J. P. Perdew, V. N. Staroverov and G. E. Scuseria, *Phys. Rev. Lett.*, 2003, 91, 146401−146401.

6. J.-D. Chai and M. Head-Gordon, *Phys. Chem. Chem. Phys.*,2008, 10, 6615–6620.
